# Exploring the Applications of Explainability in Wearable Data Analytics: Systematic Literature Review

**DOI:** 10.2196/53863

**Published:** 2024-12-24

**Authors:** Yasmin Abdelaal, Michaël Aupetit, Abdelkader Baggag, Dena Al-Thani

**Affiliations:** 1 College of Science and Engineering Hamad Bin Khalifa University Doha Qatar; 2 Qatar Computing Research Institute Hamad Bin Khalifa University Doha Qatar

**Keywords:** explainable artificial intelligence, XAI, wearable, machine learning, deep learning, health informatics, wearable sensors, user experience, wearable data, analytics, interpretation

## Abstract

**Background:**

Wearable technologies have become increasingly prominent in health care. However, intricate machine learning and deep learning algorithms often lead to the development of “black box” models, which lack transparency and comprehensibility for medical professionals and end users. In this context, the integration of explainable artificial intelligence (XAI) has emerged as a crucial solution. By providing insights into the inner workings of complex algorithms, XAI aims to foster trust and empower stakeholders to use wearable technologies responsibly.

**Objective:**

This paper aims to review the recent literature and explore the application of explainability in wearables. By examining how XAI can enhance the interpretability of generated data and models, this review sought to shed light on the possibilities that arise at the intersection of wearable technologies and XAI.

**Methods:**

We collected publications from ACM Digital Library, IEEE Xplore, PubMed, SpringerLink, JMIR, Nature, and Scopus. The eligible studies included technology-based research involving wearable devices, sensors, or mobile phones focused on explainability, machine learning, or deep learning and that used quantified self data in medical contexts. Only peer-reviewed articles, proceedings, or book chapters published in English between 2018 and 2022 were considered. We excluded duplicates, reviews, books, workshops, courses, tutorials, and talks. We analyzed 25 research papers to gain insights into the current state of explainability in wearables in the health care context.

**Results:**

Our findings revealed that wrist-worn wearables such as Fitbit and Empatica E4 are prevalent in health care applications. However, more emphasis must be placed on making the data generated by these devices explainable. Among various explainability methods, post hoc approaches stand out, with Shapley Additive Explanations as a prominent choice due to its adaptability. The outputs of explainability methods are commonly presented visually, often in the form of graphs or user-friendly reports. Nevertheless, our review highlights a limitation in user evaluation and underscores the importance of involving users in the development process.

**Conclusions:**

The integration of XAI into wearable health care technologies is crucial to address the issue of black box models. While wrist-worn wearables are widespread, there is a notable gap in making the data they generate explainable. Post hoc methods such as Shapley Additive Explanations have gained traction for their adaptability in explaining complex algorithms visually. However, user evaluation remains an area in which improvement is needed, and involving users in the development process can contribute to more transparent and reliable artificial intelligence models in health care applications. Further research in this area is essential to enhance the transparency and trustworthiness of artificial intelligence models used in wearable health care technology.

## Introduction

### Background

Wearable technologies have become indispensable and dominant in the health care landscape [1]. A notable recent shift in their use involves leveraging their capabilities for the continuous monitoring of users, which proves particularly beneficial in patient care scenarios, such as monitoring patients with diabetes to preempt hypertension [2] or in the case of athletes, analyzing heart rate (HR) data to tailor personalized exercise regimes for enhanced progress [3]. However, to develop such sophisticated models, machine learning (ML) or deep learning (DL) algorithms are used, which often function as “black boxes,” lacking transparency and making them challenging for medical professionals and end users to comprehend. In this context, explainability becomes crucial in ensuring the responsible and ethical use of wearable technologies in health care. By providing transparent insights into the inner workings of complex algorithms, explainable artificial intelligence (XAI) empowers medical professionals and end users to trust and confidently use these technologies for improved patient outcomes and personalized interventions [4]. This bridges the gap between ML experts and health care professionals, empowering the latter with actionable insights derived from these models.

Despite the growing interest and research on explainability in 2017, the association between artificial intelligence (AI) and explainability dates back to the mid-80s [5-7]. Over time, the significance of explainability has grown, recognizing its immense potential across various domains. In 2018, to further promote the importance of explainability, organizations such as the Association for Computing Machinery issued statements emphasizing algorithmic transparency and accountability [8], encouraging researchers and institutions to prioritize explainability when designing AI systems. International institutes such as the Defense Advanced Research Projects Agency have also contributed to the focus on explainability by funding the Explainable AI (XAI) Program [9]. This initiative emphasizes the importance of transparency and interpretability in AI systems, further highlighting the growing recognition of explainability’s significance in the field of AI. As a result of this, there has been a notable increase in research on explainability, emphasizing its growing importance in promoting the ethical use of AI in various domains.

In parallel, the recent emphasis on XAI has sparked interest in integrating it with wearable technologies. Wearables have demonstrated significant potential and effectiveness in health monitoring, paving the way for innovative health care applications. What sets XAI apart when applied to wearable data compared to other datasets lies in the unique characteristics of wearables. Wearables capture highly personalized, granular data, often in dynamic real-world settings. This personal and real-time nature introduces complexities that demand a specialized XAI approach. The interpretability and transparency of AI models become even more critical as users must understand not just the decisions but also their impact on health and well-being. Moreover, wearables often integrate diverse data types, from physiological signals to activity tracking, requiring XAI techniques capable of handling multimodal data. Thus, delving into XAI within the domain of wearables is essential to address these distinct challenges and harness the full potential of wearable technology in health care, fitness, and personal well-being. However, the incorporation of XAI techniques into wearables remains an emerging research frontier. A recent review, which analyzed papers from 2011 to 2022, underscored an existing gap in the field: the lack of XAI research specifically focused on interpreting 1D biosignals obtained from wearable devices [10]. To address this gap, this paper aimed to review the recent literature, exploring the application of explainability in wearables. By examining how XAI can enhance the interpretability of generated data and models, this review sought to shed light on the possibilities that arise at the intersection of wearable technologies and XAI.

### Related Studies

In the current AI era, a notable transformation is being witnessed in health care [1]. Various applications are powered by AI systems, leading to the emergence of ML and DL. As AI complexity increases, the demand for enhanced transparency is being recognized. This demand is met by the implementation of XAI, which allows AI model workings to be understood. The following paragraphs provide a more comprehensive exploration of the terminology associated with AI, ML, DL, and XAI. Following this, the paper proceeds to delve into specific research questions (RQs) that will be addressed.

AI involves the creation of systems and machines designed to replicate human intelligence, enabling them to perform real-world tasks effectively. AI systems are trained on data, allowing them to learn from experience and solve specific problems. They continuously refine their performance based on the information they receive. AI applications are diverse and include advanced web search engines, self-driving cars, gaming, speech recognition, recommendation systems, and health care AI systems and applications. AI essentially emulates human cognitive processes, making it invaluable when dealing with extensive datasets [1].

ML, a subset of AI, empowers computers to recognize patterns, make highly accurate predictions, and self-improve through experiential learning without the need for explicit programming. Building AI-driven applications relies heavily on ML methodologies. These models undergo training using extensive datasets, enabling them to deliver precise predictions [2]. ML is further categorized into supervised and unsupervised learning, with supervised learning branching into semisupervised learning [4] and reinforcement learning [5].

**Figure 1:**
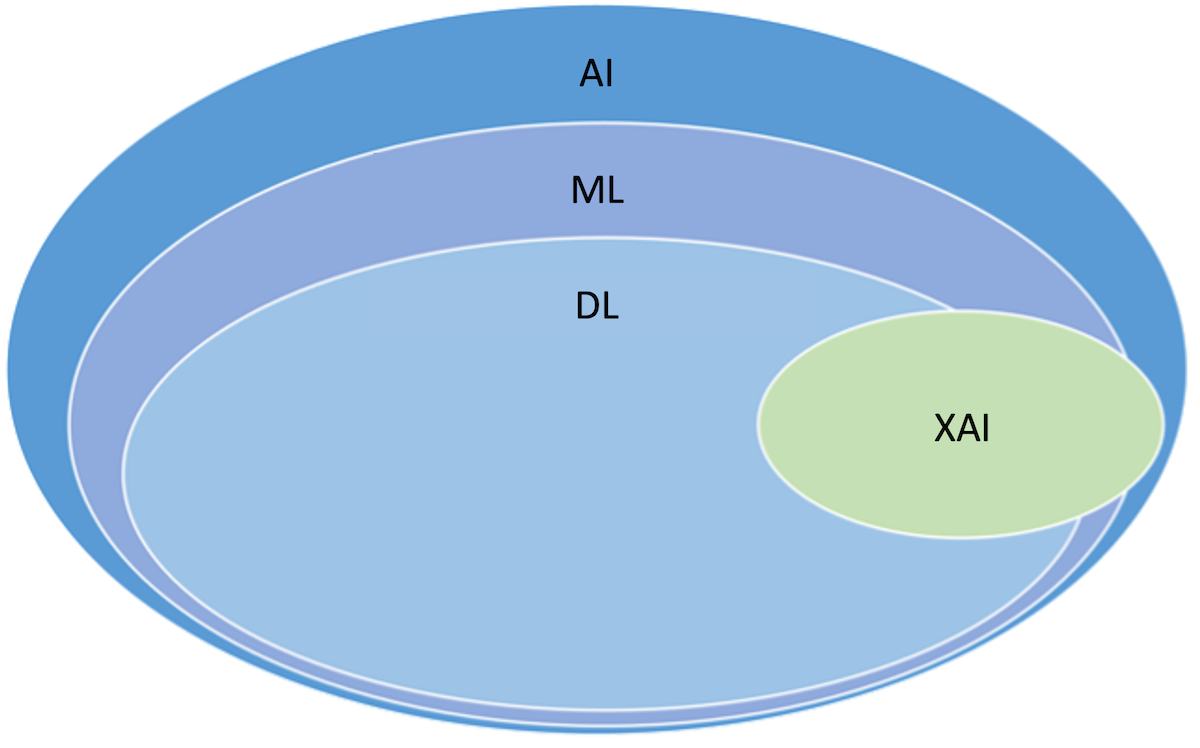
Relationship among artificial intelligence (AI), machine learning (ML), deep learning (DL), and explainable AI (XAI) [13].

DL, a specialized field within ML, draws inspiration from the human brain’s structure and functionality. DL effectively uses both structured and unstructured data for model training. It plays a vital role in predicting life-threatening diseases in medical research, with deep neural networks achieving remarkable predictive capabilities [11]. Prominent DL models include convolutional neural networks (CNNs), residual networks, fully convolutional networks, and long short-term memory (LSTM) [12]. Figure 1 [13] depicts the relationship among AI, ML, DL, and XAI.

XAI enriches AI models with information comprehensible to the end users. While AI algorithms enable users to make informed business decisions, the opacity of these algorithms often leaves users uninformed about the decision-making processes [14]. This lack of transparency is where XAI comes into play. XAI strives to elucidate the inner workings of AI models, offering users comprehensible explanations of the methodologies, procedures, and outputs. The term *XAI* is often referred to as the “white box” approach due to its emphasis on revealing the model’s processes.

**Figure 2:**
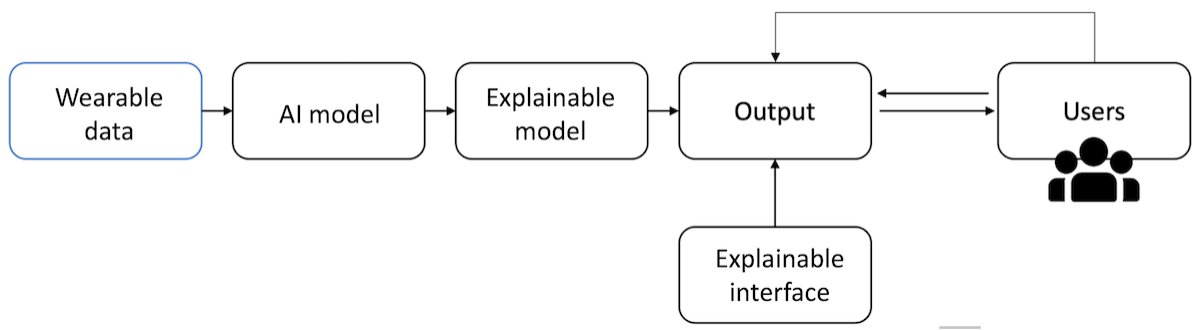
Process of explainable artificial intelligence (AI) from wearable data, adapted from Saranya and Subhashini [13].

In the field of XAI, training data serve as input, and users select the prediction methodology and XAI techniques based on specific application requirements. The input data vary depending on their source. For example, they can be electronic health records, vital sign recordings, medical scans, and wearable data. This review focuses on wearable data as the training data. Wearable data vary among physiological, activity, environmental, behavioral, biometric, and social interaction data. These techniques shed light on the model’s internal operations and provide an explanatory interface, as shown in Figure 2, adapted from the study by Saranya and Subhashini [13]. In the modified diagram, the input data are changed to “wearable data” to be more specific to this review. This transparency empowers users with insights into AI model outputs, fostering trust [15]. Armed with this understanding, users can enhance output accuracy and identify model shortcomings, facilitating informed decision-making to improve the model.

Despite the recent surge in wearable devices within the health care sector, there is a notable gap in research on XAI applied to wearable data. The application of XAI in wearable data analysis is crucial due to several key factors. First, the high granularity and personal nature of the data collected via wearables emphasize the need for transparency. Users must understand how their data are processed and interpreted to trust the insights generated. Second, given the inherently personal nature of wearables, establishing trust becomes paramount. Users need confidence in the accuracy and privacy of the data collected, making transparency in algorithms and data handling essential. Finally, wearables operate in dynamic environments and integrate diverse data types. XAI can help unravel complex relationships within these data streams, enabling better insights and decisions in various health care and well-being applications. Although recent reviews have explored the broader applications of XAI in health care, the potential of wearables in this context has remained somewhat overshadowed. Some of these reviews have touched on the relevance of wearable biosensors in health care applications, yet they often fail to fully uncover the diverse opportunities presented by both commercial and noninvasive smartwatches [16,17]. Similarly, certain reviews have focused on health care Internet of Things devices, but they tend to only scratch the surface regarding the intricate field of wearable technology [18,19]. The limited number of studies on XAI from wearable data highlights a significant research gap in this emerging field. While wearables have gained traction in health care and other domains for data collection and analysis, the incorporation of XAI principles to ensure the interpretability of AI models is an area that holds great potential but has seen limited exploration. This review aimed to explore the use of XAI within wearables (RQ 1).

In the domain of wearable technology, these devices offer a diverse array of data, encompassing activity metrics such as calorie expenditure and step counts as well as physiological signals such as electrodermal activity (EDA) and electrocardiography (ECG). Furthermore, wearables extend their capabilities to encompass behavioral patterns and environmental cues, thereby enriching the contextual understanding derived from the data they collect. Recent reviews in the health care field have illuminated the widespread adoption of wearables across various anatomical regions, including the head, limbs, and torso [20]. These versatile devices have been instrumental in monitoring a range of medical conditions, spanning stroke and poststroke rehabilitation to Parkinson disease, among others [21]. Hence, the adoption of wearables in health care is evident, yet some studies have delved deeper and adopted XAI for wearable data. For example, a study used EDA for pain recognition using the gradient-weighted class activation mapping technique [22], and another adopted accelerometer data for fall detection using local interpretable model-agnostic explanations (LIME) as the explainability method [23]. This review sought to address the question of which specific wearable data types have been explored within the context of explainability (RQ 2). By shedding light on this aspect, this review sought to bridge the gap and provide insights into the areas of wearable data that warrant greater attention from researchers and practitioners in the field of XAI. In addition, this review sheds light on the various explainability methods deployed specifically for each type of wearable data (RQ 3).

Previous reviews on XAI in the health care field have focused on explainability. They have highlighted the dominance of feature explanations over textual and example-based explanation methods. In addition, most explainability methods use the post hoc approach while focusing on the local rather than the global scope of the data. This review aimed to identify whether wearable data follow this trend from previous reviews (RQ 4).

The evaluation of AI models after applying an explainability method is crucial for ensuring transparency, accountability, and user understanding. Several researchers have stressed the need for formal evaluation metrics and a more systematic evaluation of the XAI methods [5,24]. Evaluation allows for a formal comparison of the available explanation methods [25] and offers a formal method to assess whether explainability is achieved in an application [25]. A previous review focused on the importance of assessing the explanation, and the results revealed that only 1 in 3 studies solely relied on anecdotal evidence for evaluation, whereas 1 in 5 studies incorporated user evaluations [26]. This highlights the gap in evaluating the XAI outcomes through either anecdotal evidence or user evaluations. To address this gap with wearable data, this review aimed to identify the evaluation methods used for the XAI techniques in the field of wearable data (RQ 5).

These aspects led to the following RQs, which guided our survey:

1. In the health care domain, how is XAI being used within the context of wearables? (RQ 1)
2. What is the predominant data type used in building XAI models from wearables? (RQ 2)
3. How is the explainability of AI models represented to the users? (RQ 3)
4. To what extent do model-agnostic, post hoc, and global explainability methods prevail compared to other approaches in line with existing literature? (RQ 4)
5. What are the evaluation methods used for various explainability techniques in the context of wearables and XAI in health care? (RQ 5)

### A Typology of XAI Features

#### Overview

To guide the exploration of XAI within the domain of wearable technology, recent reviews on explainability have provided categorizations of XAI methods. These reviews have extensively examined various dimensions of XAI. One review focused on 6 dimensions, namely, the type of explanation, type of task, type of data, type of explainability method, type of problem, and type of model to be explained [26]. Another review focused on the application domain, model type, stage, scope, and output format [16]. We opted to build our survey on those previous XAI taxonomies [27] as this informs the different forms of explainability deployed in wearable technology, encompassing stage, scope, problem type, input data, and output format. This taxonomy is recent, covers relevant concepts, and has been highly cited by other researchers.

The following sections provide a brief definition of each explainability dimension.

#### Input Data of the XAI System

The term *input data* within the context of XAI systems refers to the data used to train the AI model, as shown in Figure 2 [13]. The nature of these input data can vary based on their source. In the context of this review, which focuses on wearable data, the primary source of input data is wearable devices. Wearable data encompass various forms, including physiological signals such as HR, ECG, and electroencephalography. In addition, wearables provide data in the form of activity metrics such as step and calorie counts. Moreover, wearable technology captures behavioral and emotional states, allowing users to log factors such as stress levels. Environmental data, including temperature and ambient noise levels, can also be collected via wearables. Furthermore, wearables can capture social data, such as monitoring the time spent on specific social media apps and related interactions.

#### Output Format of the XAI System

Output format of explainability refers to how the explanations are presented. This can vary from *visual* representations such as graphs and images to *textual*, *numerical*, *rule-based*, or *mixed* formats [27]. The presentation of explainability encompasses diverse approaches that depend on several factors, such as the target population and the nature of the input data. For instance, when the target population consists of lay users, explainability must be presented in a simple manner that aligns with their level of understanding. Conversely, when explainability is intended for medical professionals or researchers, a more detailed and in-depth approach is necessary as this population possesses a higher level of expertise and requires a comprehensive understanding of the underlying mechanisms and processes driving the model’s outcomes. In addition, each type of input data may require specific methods of explanation. The nature and characteristics of the input data play a role in determining the most effective approach for conveying their insights and interpretations.

#### Stage of Explainability

Stage of explainability refers to the point during the XAI process (Figure 2 [13]) at which a method generates explanations. The stage when the explainability is introduced can be *post hoc* or *ante hoc*. Ante hoc methods aim to consider the explainability of a model from the beginning and during training to make it naturally explainable. In contrast, post hoc methods maintain pretrained models without any structural modifications and introduce explainability mechanisms after the model’s training phase. The post hoc methods frequently use external explainer techniques during testing. An example of a post hoc method is Shapley Additive Explanations (SHAP) [28], which aims to provide a way to attribute the contribution of each feature to a model’s prediction. Conversely, an example of an ante hoc method is recurrent lexicon networks [29], a method that models lexicons as naïve gated recurrent networks while seamlessly integrating explainability principles throughout the training process. This approach ensures that the model inherently possesses explainability characteristics from the beginning.

#### Scope of Explainability

The scope of explainability refers to the extent to which an explanation clarifies the inner workings of the AI model. The scope of explainability can be either local or global [30]. Local explainability focuses on clarifying the reasoning behind individual predictions, offering insights into why a specific decision was made for a particular instance. For instance, by using techniques such as LIME [31], a model may reveal that it diagnosed a rare medical condition for a patient based on a combination of relevant biomarkers and wearable data. Conversely, global explainability seeks to provide a broader perspective, offering a holistic understanding of the model’s behavior and feature importance across an entire dataset. Using methods such as SHAP [28], one can analyze the model’s tendencies in an entire patient population. Thus, local explainability delves into explaining individual predictions, whereas global explainability offers insights into the model’s behavior and feature importance across an entire dataset.

#### Problem Types Addressed by AI Models

Each AI model that is constructed is tailored to address a specific underlying problem type, which could fall into either *regression* or *classification* categories. Studies that use regression aim to predict a continuous numerical value, whereas classification focuses on categorizing data into distinct classes or groups. Regression models are valuable for predicting patient outcomes and estimating essential health parameters. For instance, these models can be used to predict blood pressure levels in patients with diabetes based on factors such as food intake, exercise, and insulin dosage [32]. On the other hand, classification models are instrumental in disease diagnosis and treatment planning. For example, ML models can analyze physiological data to classify an individual’s stress levels [33].

## Methods

### Study Design

We followed a systematic review design using qualitative methods. We adhered to the PRISMA (Preferred Reporting Items for Systematic Reviews and Meta-Analyses) statement [34].

### Data Sources and Search Strategy

At the time of conducting our study, we captured the most recent publications in the rapidly evolving fields of XAI and wearable devices. Since the Defense Advanced Research Projects Agency introduced the XAI program in 2017, there has been an increase in papers focusing on XAI. Recent reviews have shown a significant rise in publications in the field of XAI during the period from 2018 to 2022, highlighting the growing research interest and developments [13] in the field, and this trend continues today. In this review, we collected publications covering 3 years, from January 1, 2018, to December 31, 2022, and then conducted the analysis and reporting process. We deem the qualitative outcomes of our review also representative of more recent publications in the field. We collected the publications from ACM Digital Library, IEEE Xplore, PubMed, SpringerLink, JMIR, Nature, and Scopus. The selected publishers are renowned for their high-quality and impactful research across computer science, engineering, health care, and interdisciplinary studies. In addition, our preliminary search indicated the potential of these publishers to provide research on XAI and wearable data. The search strategy encompassed a comprehensive range of terms from various domains. These included explainability concepts, AI (Medical Subject Heading; MeSH) terminology, target population (MeSH) descriptors, wellness (MeSH) terms, and technology (MeSH) keywords. XAI, ML, and DL were considered alongside target populations such as patients and physicians. Wellness-related terms such as *physical activity*, *sleep*, and *exercise* were also incorporated. In addition, technology aspects encompassed wearable electronic devices and sensors. Multimedia Appendix 1 provides detailed keywords for each database.

### Eligibility Criteria

The inclusion criteria for this review encompassed technology-based research involving wearable devices, sensors, and mobile phones. The studies were required to incorporate explainability or ML or DL techniques and use quantified self data. The quantified self domain, which began in 2007, uses technology such as apps and wearable smart devices to monitor, measure, and quantify different aspects of daily life [35]. In addition, the focus of the studies needed to be medical, and they had to be proceedings, book chapters, or peer-reviewed journals; written in English; and published between 2018 and 2022. On the other hand, the exclusion criteria included duplicate studies, review articles, and books, as well as workshop papers, courses, tutorials, and talks. Textbox 1 provides detailed information.

**Inclusion and exclusion criteria.**

**Inclusion criteria**

- Technology-based research (eg, wearable devices, sensors, and mobile phones)
- Studies incorporating explainability
- Studies incorporating machine learning or deep learning techniques
- Studies using quantified self data
- Medical-based studies
- Studies published in peer-reviewed journals
- Proceedings or book chapters
- Studies written in English
- Studies published from 2018 to 2022

**Exclusion criteria**

- Duplicate studies and review articles
- Books
- Workshop papers
- Courses
- Tutorials
- Talks

### Study Screening

Screening of potentially eligible studies was performed in 3 steps: duplicate removal, title and abstract screening, and full-text screening. Duplicates were removed using Rayyan (Rayyan Systems Inc) [36]. Additional duplicates that were not removed during this process were removed manually. Two review authors (YA and MA) independently screened the titles and abstracts for inclusion using the predefined inclusion and exclusion criteria specified previously. The other 2 review authors (DA-T and AB) independently screened a random 30% (n=197) of the included studies. Agreement among all authors was confirmed using a Cohen κ test. Table 1 provides more details.

**Table 1:** Cohen κ test of reviewer agreement.

Author initials		YA	DA-T	MA	AB
**YA**					
	Cohen κ	—^a^	0.63	1	0.78
	Percentage of agreement	—	94.17	100	96.58
**DA-** **T**					
	Cohen κ	0.63	—	0.63	0.63
	Percentage of agreement	94.17	—	94.17	94.17
**MA**					
	Cohen κ	1	0.63	—	0.78
	Percentage of agreement	100	94.17	—	96.57
**AB**					
	Cohen κ	0.78	0.63	0.78	—
	Percentage of agreement	96.58	94.17	96.57	—

^a^Not applicable.

All studies not discarded through this process were then screened by 1 review author (YA) in a full-text review process from which studies were identified for inclusion. Subsequently, a backward and forward referencing approach was used to further uncover potentially relevant papers. Conflicts among the 4 review authors were addressed through a majority vote system where a 3-versus-1 decision was made. Only 3 papers were found to have 2-versus-2 conflicts, which were subsequently resolved through discussions with each review author. Full data extraction, categorization, and labeling of papers were performed by 1 author (YA) and validated by 2 authors (MA and AB).

### Feature Extraction

During the analysis, various features were extracted from different perspectives. *Metadata features* provided information about the publication and dissemination of the selected studies. *Explainability features* delved into the specific problem, the AI model used, and the input data used, following the taxonomy [27]. *Evaluation of explainability* included whether it was evaluated through user studies. For studies involving user evaluations, additional features such as the materials used, participant count, data collected, and outcome of the evaluation were extracted. *Technology features* focused on the type of wearable device used and its placement on the body. Table 2 provides detailed information. All features except the explainability features were created by the authors to fit the topic.

**Table 2:** Feature extraction.

Viewpoint and feature		Description
**Metadata (PRISMA Flowchart and Selection Statistics and Geographic and Time Statistics sections)**		
	Country	Country of the article based on the affiliation of the first author
	Domain	Targeted domain of the study, such as a specific disease or medical condition (eg, diabetes or stress management)
	Publication date	When the article was published
**Explainability [27] (Analysis Using the Typology of XAI Features section)**		
	Model	Type of ML^a^ or DL^b^ model that was used for performing the primary task
	Problem type	Methods for explainability can vary according to the underlying problem (classification or regression)
	Output format	Form of explanation generated for the model’s outcome
	Target group	The group targeted by the explainability generated
	Input data	Type of quantified self data used, such as step count or calories
	Stage	Refers to the stage at which a method generates explanations; can be either ante hoc or post hoc
	Scope	Refers to the scope of an explanation; can be either global or local
**Evaluation** **(Human-Centered Evaluation section)**		
	Evaluation method	Method of evaluation; can be with or without end users
	Study design	The study design used for verifying the explainability
	Study method	The method used, such as qualitative, quantitative, or mixed methods
	Participants for building the model	Number of participants recruited for collecting data to build the model
	Participants for testing the model	Number of participants recruited for collecting data to test the model
	Type of participants	Participant types, such as healthy or disease or condition specific
	Data collection methods	Methods used to collect the data for the user study
	Duration of the study	Duration of the user study
	Medium of interaction	Medium used to communicate the explainability
	Study procedure	The intervention of the user study
	Data analysis	Techniques used for analyzing the user study
**Technology** **(Technologies for Capturing Quantified Self Data section)**		
	Type of technology	Different types of wearable technology used, such as Empatica E4 wristband or Fitbit wearables
	Position of the wearable	Position of the wearable device, such as on the wrist or arm

^a^ML: machine learning.

^b^DL: deep learning.

### Data Synthesis and Analysis

We used descriptive statistics to describe the metadata, explainability features, human-centered evaluation, and technology used.

## Results

### PRISMA Flowchart and Selection Statistics

**Figure 3:**
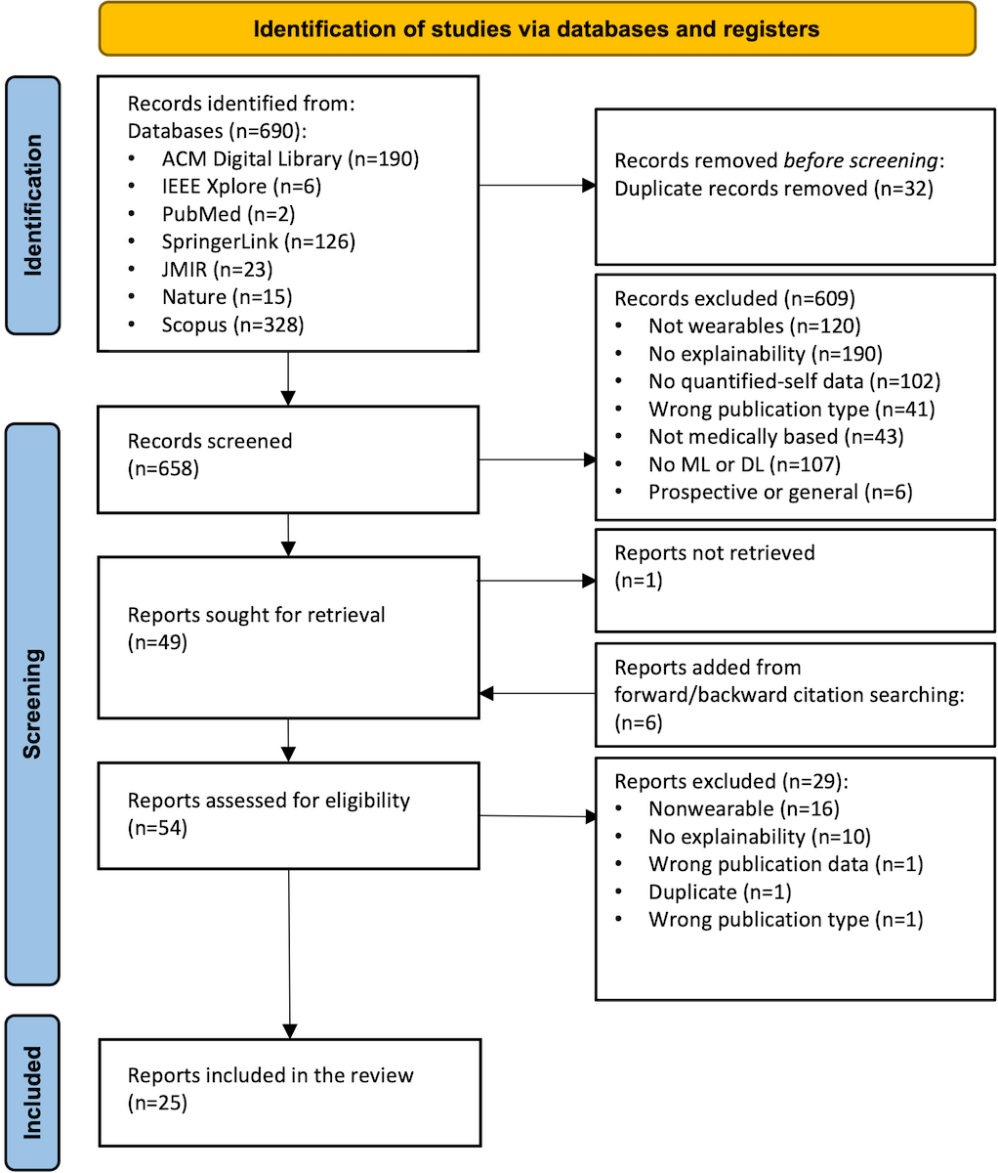
PRISMA (Preferred Reporting Items for Systematic Reviews and Meta-Analyses) flow diagram of the systematic review. DL: deep learning; ML: machine learning.

The study selection sequence is outlined in a PRISMA flowchart, which is shown in Figure 3. Our search yielded 690 articles, of which 32 (4.6%) were identified as duplicates and removed. After screening abstracts, a further 609 (88.3%) were excluded, leaving 49 (7.1%) assessed for eligibility via full-text review. Among these, 29 (59.2%) were excluded. Forward and backward citation searching yielded an additional 6 included papers. A total of 25 studies met the inclusion criteria after completing the backward and forward citation check. Refer to Multimedia Appendix 2 for the PRISMA checklist.

### Geographic and Time Statistics

**Figure 4:**
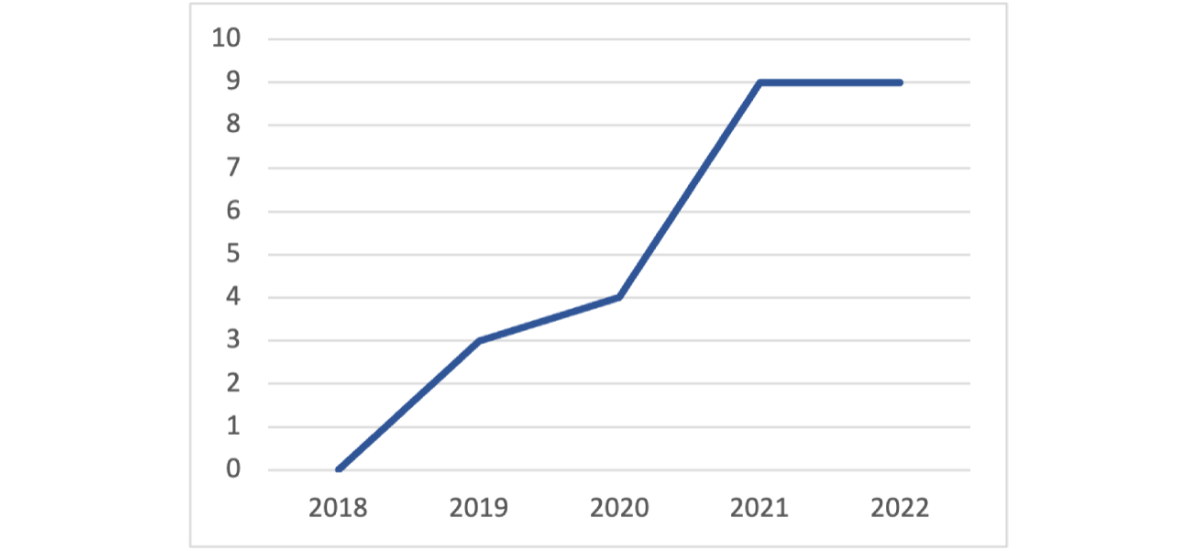
Distribution of included papers per year.

Table 3 illustrates the distribution of the 25 included papers, with 10 (40%) originating from the United States, 8 (32%) originating from various European countries, 1 (4%) originating from China, and 6 (24%) originating from South Korea. This widespread interest and research focus on explainability in wearable devices indicates its global significance and relevance in diverse regions. Furthermore, Figure 4 highlights that the selected papers were primarily published between 2019 and 2022, indicating a recent uptrend in research activity in this field. This recent attention highlights the growing recognition and importance of integrating explainability with wearable technologies for advancing health care applications.

**Table 3:** Distribution of studies per country (N=25).

Country	Publications, n (%)
United States	10 (40)
South Korea	6 (24)
The Netherlands	2 (8)
Switzerland	2 (8)
China	1 (4)
Italy	1 (4)
Norway	1 (4)
Turkey	1 (4)
United Kingdom	1 (4)

### Application Domains

In this systematic review, certain domains received more exploration and attention in the context of explainability in health care monitoring compared to others, as shown in Table 4. When assessing the depth of investigation, certain domains stand out, with health conditions and diseases being the most extensively studied, accounting for 24% (6/25) of the research focus. Notably, these studies delved into conditions such as kidney disease, multiple sclerosis, influenza, sarcopenia, osteopenia, and the ongoing COVID-19 pandemic. Similarly, sleep and activity monitoring attracted some research interest, constituting 24% (6/25) of the reviewed studies. These investigations provided valuable insights into individuals’ sleep patterns, personal health, and activity levels. Vital sign and health monitoring, encompassing parameters such as blood pressure and blood glucose levels, shared a comparable portion of the research landscape, also constituting 24% (6/25) of the total. This attention stems from the critical role of vital sign and health monitoring in the early detection and management of various health conditions, including diabetes. In comparison, domains such as mental health, stress management, and weight management collectively accounted for 16% (4/25) of the research focus. While these areas received some attention, they stood slightly behind the aforementioned domains. Finally, domains such as activity recognition, neurological monitoring (specifically brain signals), and substance abuse detection with a focus on opioid detection collectively constituted 12% (3/25) of the reviewed papers. These domains, while important, received relatively less exploration within the context of explainability in health care monitoring.

The explainability of wearable devices was applied in various health domains. It was widely used in the physical activity and health domain, such as predicting user-specific health risks [35] and identifying effective representations of fitness goals to enhance user physical activity and trust in the system. Wearable devices were also extensively used for diabetes control. For example, some studies (2/25, 8%) used the wearable device Empatica E4 and glucose monitoring to detect hypoglycemia [37] and hyperglycemia [2] with a lead time of up to 60 minutes. Another study focused on detecting eating moments and explaining glucose levels using wearables [32]. Similarly, other studies (2/25, 8%) focused on blood pressure monitoring and generated personalized lifestyle recommendations based on the user’s blood pressure [38,39]. It is worth noting that there were not many studies exploring XAI for weight management [40]. This is interesting because it could have practical applications for a large user base, including nonexperts. One possible reason for this gap in research might be that weight-related data are relatively easy to understand for most people and something that can be easily measured. In contrast, more complex data types received more attention in the field of XAI.

**Table 4:** The different wearable data applications using explainability.

Applications	Studies, n (%)
Vital sign and health monitoring	6 (24)
Sleep and activity monitoring	6 (24)
Health conditions or diseases	6 (24)
Mental health	3 (12)
Opioid abuse and detection	1 (4)
Weight management	1 (4)
Neurological and brain signals	1 (4)
Human activity recognition	1 (4)

Stress detection and management also received attention in wearable research. A study focused on stress detection and coping strategies [33], whereas another explored the prediction of next-day perceived stress using physiological signals such as ECG [41]. Comparative studies were also conducted to compare the effectiveness of different wearables in stress detection [42]. Furthermore, wearables were used in medical applications such as opioid detection [35] and identifying COVID-19 [43]. These studies highlight the diverse applications and potential of explainable wearable devices in health care.

### Analysis Using the Typology of XAI Features

#### Overview

**Figure 5:**
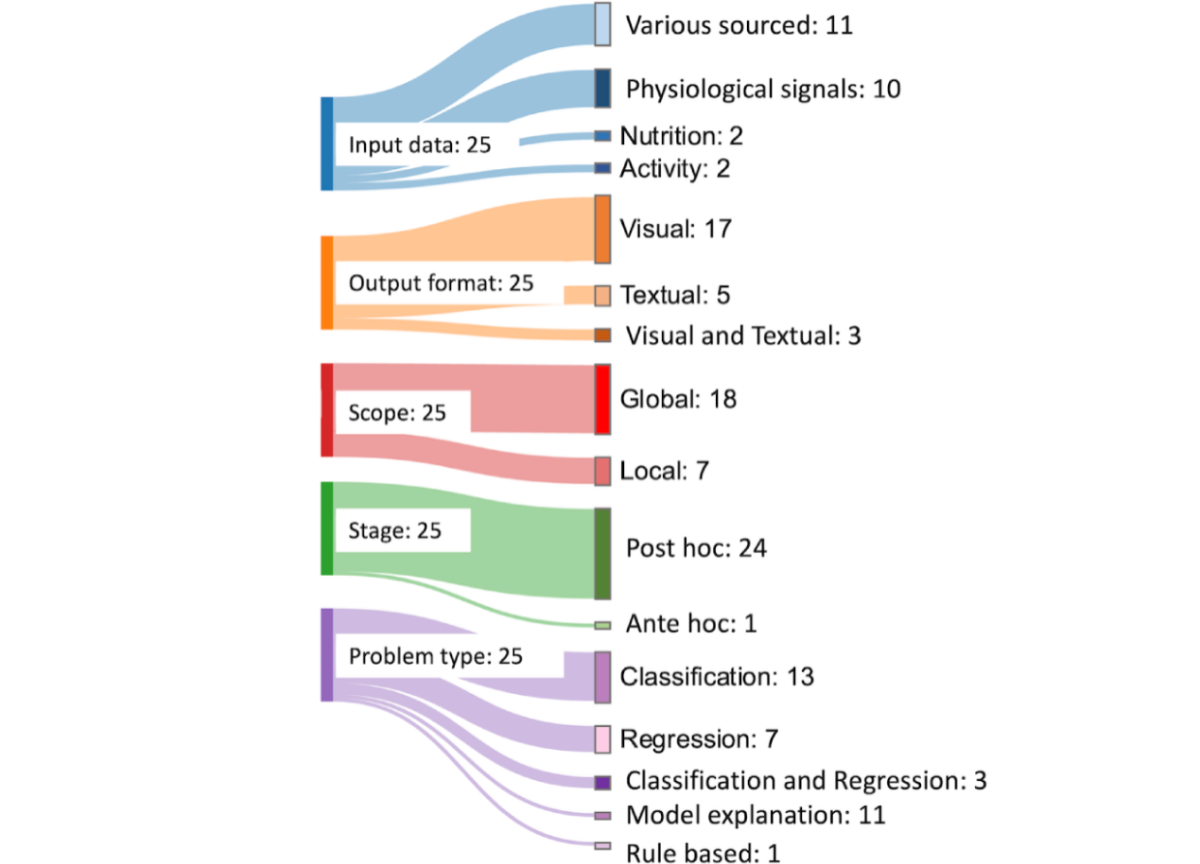
Overview of the explainability features.

The following sections aim to provide concise descriptions of the primary categories of explainability methods identified in this systematic review (Multimedia Appendix 3 [2,3,33,38-53]). This is followed by a summarization of their stage, scope, problem type, input data, and output format. Figure 5 provides a summary of the 25 included articles, categorizing them into the five explainability features identified in the study.

#### Input Data of the XAI System

**Figure 6:**
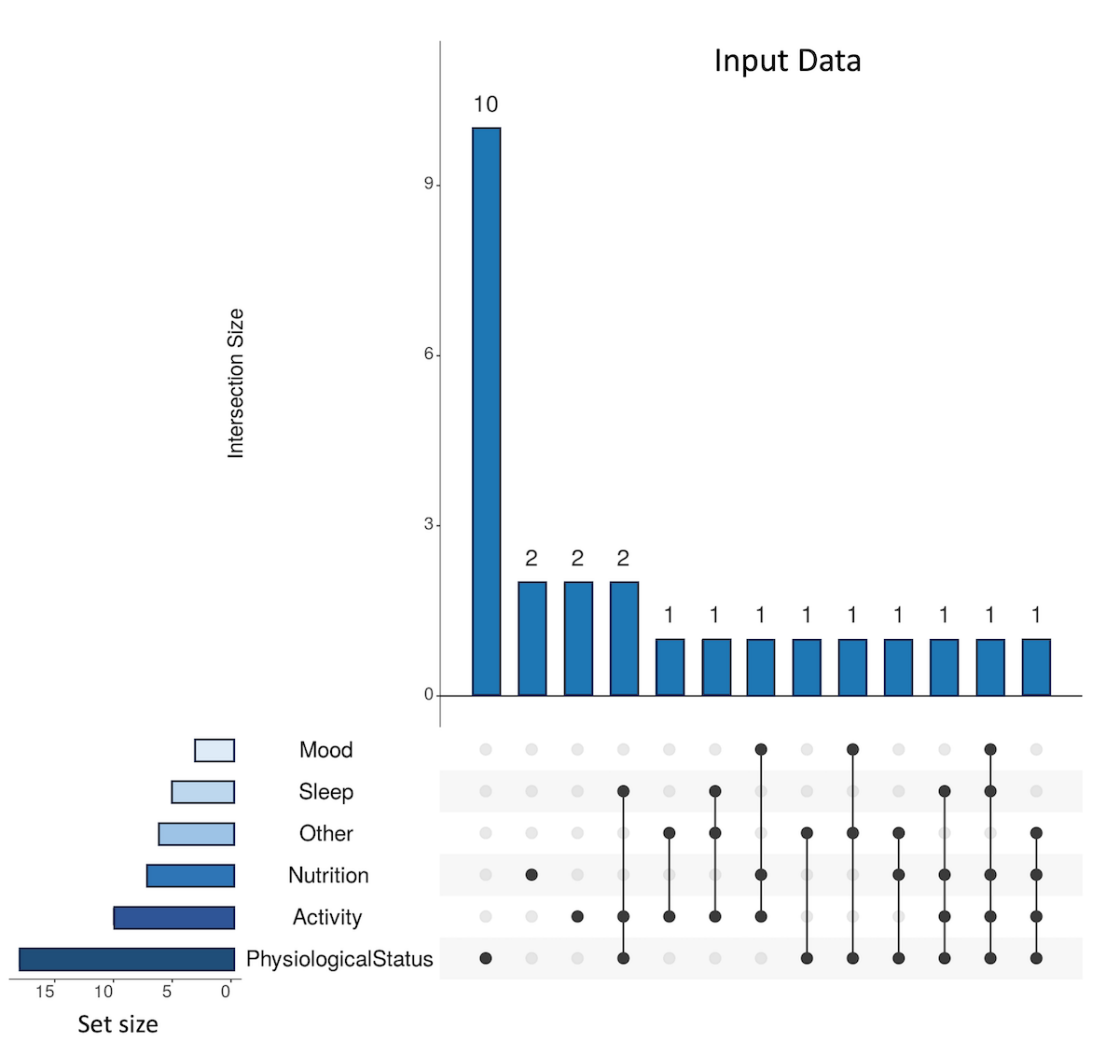
UpSet diagram [45,46] of the various combinations of input data.

This systematic review identified 5 primary categories of input data used in the research, including physiological signals (eg, HR), activity data (eg, steps), sleep data (eg, sleep duration and stages), nutritional data (eg, calorie intake), and mood (measured through surveys). Among these categories, physiological signals emerged as the most used input data for developing AI models, as shown in Figure 6 [44,54]. This prevalence can be attributed to the widespread use of wearable technology for collecting quantified self data. Commercial wearables such as Fitbit and Apple Watch excel in capturing physiological information such as HR, whereas medical wearables such as Empatica E4 offer advanced capabilities for gathering signals such as EDA, photoplethysmography, HR variability, and accelerometer data. Several studies in the reviewed literature (3/25, 12%) opted for a multisource approach, using various input data types to obtain a comprehensive understanding of the user’s health, as shown in Figure 6 [44,54]. For instance, researchers incorporated a combination of physiological signals, activity data, and sleep metrics into their analyses [43].

The input data in this review can also be classified into manual and automatic data collection methods, as shown in Textbox 2. Automatic data collection involves using technological devices such as wearables or mobile apps to gather data without direct user involvement, whereas manual data collection requires users to actively provide information or respond to specific queries. For instance, nutritional data are manually entered by users to estimate calorie intake [32], and mood data are collected through questionnaires to identify stress levels [33]. By considering both manual and automatic data collection, researchers can obtain a comprehensive and diverse set of input data, leading to a more holistic understanding [39]. Automatic data collection occurs with devices such as continuous glucose monitoring systems, which continuously measure and record glucose levels. Some studies in this review (3/25, 12%) used devices such as Freestyle Libre for blood glucose monitoring [2,32,37].

**Categorization of input data into automatically and manually captured.**

**Automatic (sensor signals)**

- Physiological signals
- Activity
- Mobility
- App use
- Environmental data
- Sleep

**Manual (logs)**

- Weight
- Food intake
- Mood

#### Output Format of the XAI System

#### Overview

**Figure 7:**
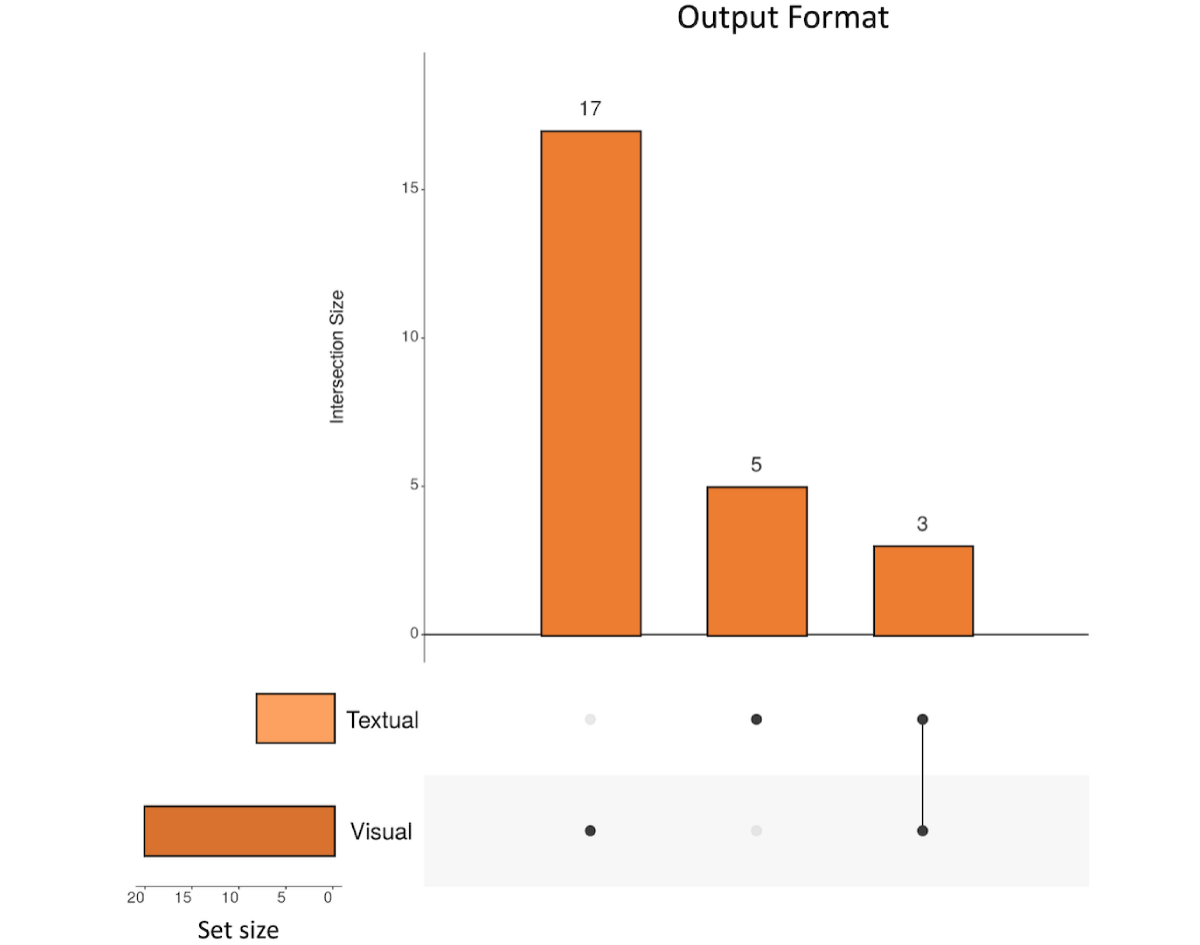
Overview of the explainability formats used in the studies included in this review.

The following sections delve into the various explainability methods found in this review. Figure 7 summarizes the various output formats in this review. Among the reviewed studies, 64% (16/25) primarily used visualizations for explanations, whereas 16% (4/25) relied solely on text. A total of 12% (3/25) of the studies combined visual and textual explanations, and only 4% (1/25) used rule-based explanations, highlighting the diversity in interpretability approaches for health care–monitoring AI models.

#### Visual Explanations

Visual explanations provide a natural and engaging means of conveying information and are highly effective in facilitating understanding. They are particularly useful in explaining complex concepts or processes as they can leverage the power of visual imagery to enhance comprehension. In the context of explainability, visual explanations can play a crucial role in explaining the “black-box” models in simpler means. Figure 7 shows the prevalence of visual explainability methods, highlighting their significance in the field. These methods use graphical tools and visual representations to provide insights into how a model operates and arrives at its predictions or decisions.

**Figure 8:**
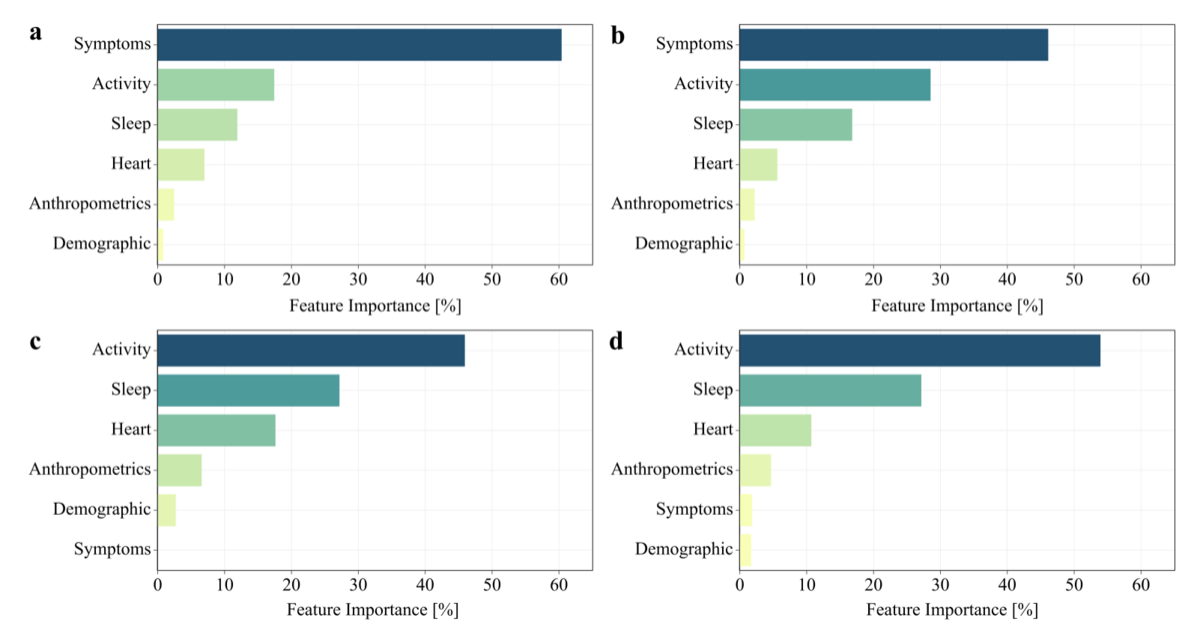
Explainable gradient boosting of COVID-19 symptoms using bar charts [43].

**Figure 9:**
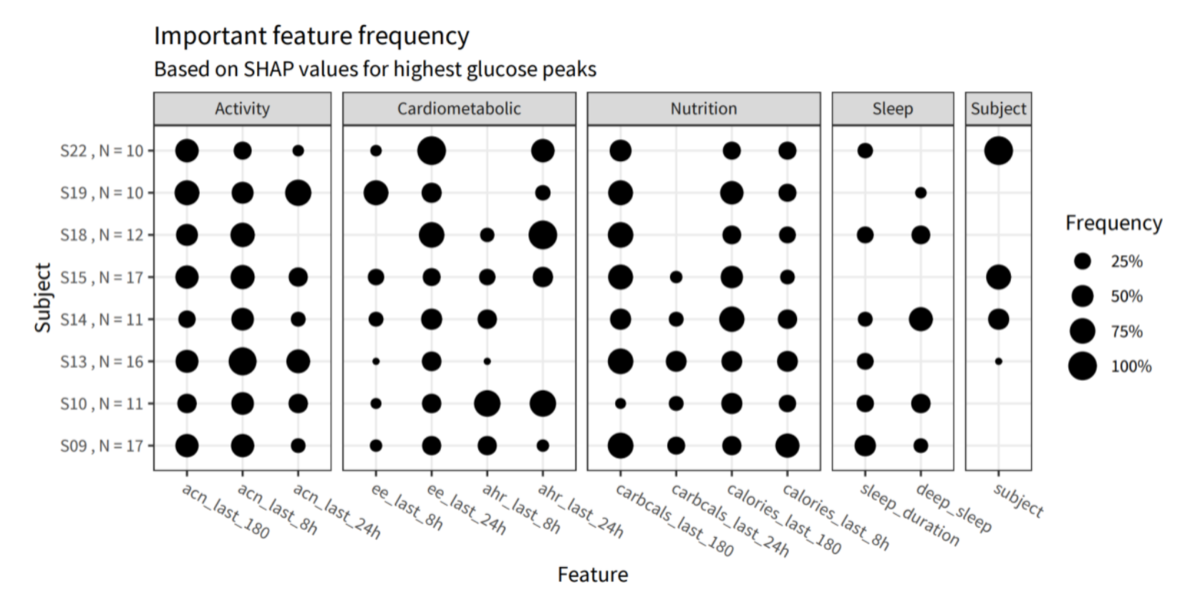
Shapley Additive Explanations explainability using a bubble frequency plot [32].

In some cases, additional visual aids such as graphs and scatter plots are used to generate visual explanations. For instance, a study focusing on COVID-19 detection used a gradient boosting prediction model based on decision trees. To illustrate the importance of different variables in the detection process, a bar plot was used, allowing researchers to visually identify the feature significance, as shown in Figure 8 [43]. Similarly, frequency bubble plots were used in certain studies to visualize glucose levels in healthy individuals, as shown in Figure 9 [32]. This visual representation assists in supporting individuals’ self-management of their health by providing clear and easily interpretable information about their glucose levels [32]. Overall, visual explanations offer a powerful and accessible approach to communicating complex concepts, system behavior, and feature importance, enabling better understanding and informed decision-making.

#### Textual Explanations

**Figure 10:**
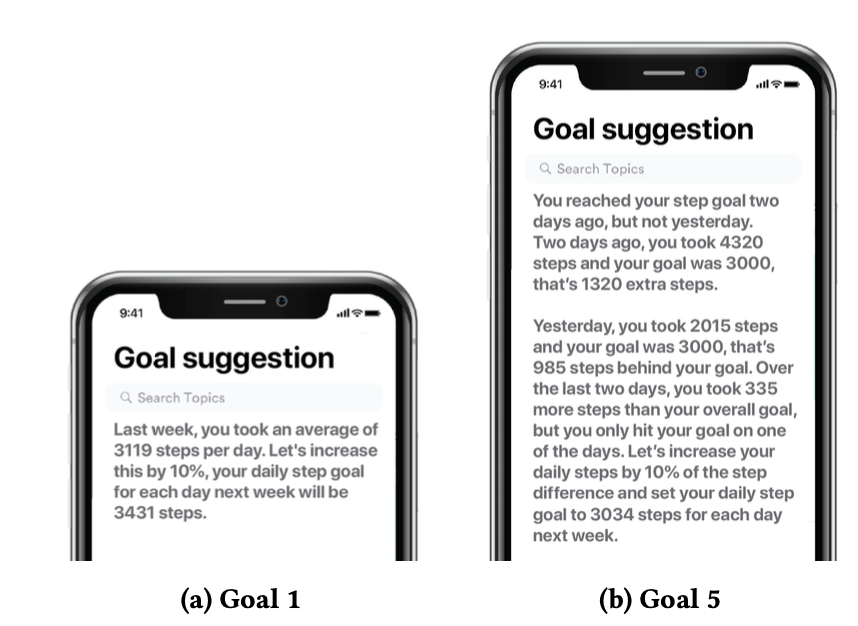
Fitness tracker goals displayed as plain text [47].

Textual explanations are another intuitive and widely used method for providing explanations. They are presented as natural language statements, whether written or spoken. Figure 10 [55] shown the significant use of text in the explainability of AI models. In various studies (3/25, 12%) [39,55-57], textual explanations enhanced transparency and trust in different domains.

For example, one study compared 2 textual formats for suggesting fitness tracker goals, focusing on improving the transparency of step goal computation, thereby fostering user–AI model understanding and trust [55]. Furthermore, textual explanations were used to generate comprehensive summaries of personal health data, enhancing their explainability [56]. These summaries involve generating natural language descriptions of temporal trends and patterns from time-series health data. Such summaries help users evaluate their health data and compare them to their goals or general health guidelines. In addition, pattern-based summaries help identify hidden behaviors and provide explanations to the user. A linguistic summarization approach leveraging time-series protoforms was used to generate these summaries [56]. Protoforms act as sentence templates with placeholders automatically selected to reflect trends and patterns supported by the data.

#### Multimodal Explanations

**Figure 11:**
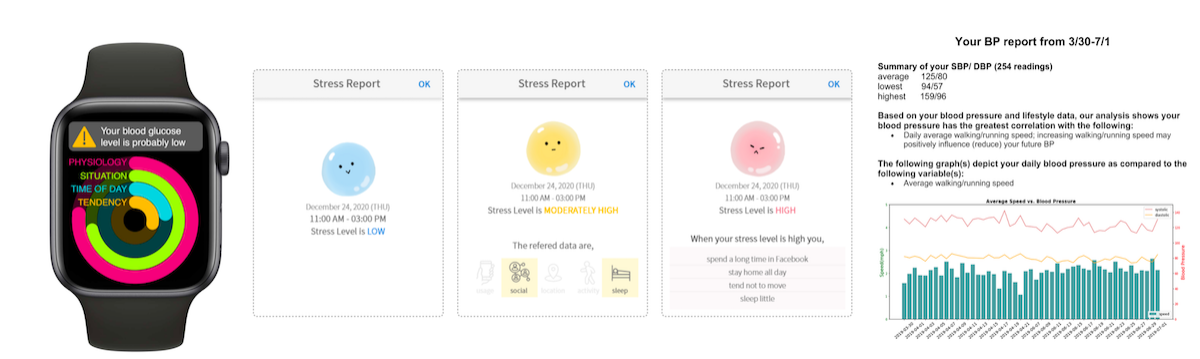
(A) Stress-monitoring app [37] using a ring chart; (B) detecting hypoglycemia using wearables [33] displayed as text and emoticons; (C) blood pressure monitoring and lifestyle recommendations given as plain text and bar and line charts [38].

In explainability research, tailoring the presentation to end users is crucial for effective comprehension and engagement. One approach involves leveraging familiar and user-friendly graphics to convey information in an accessible manner. Figure 11A [37] shows the use of activity rings from the Apple Watch, which are already well known to users [39]. In the context of hypoglycemia detection for patients with diabetes, the nearly closed violet ring in the visual representation serves as a warning sign for low blood glucose levels. This intuitive approach incorporates the individual’s physiological state as a significant factor in identifying hypoglycemia.

Similarly, when developing an app for stress prediction, it is essential to ensure that end users can easily interpret the results of the AI model. Visual representations prove valuable in illustrating the level of stress experienced by the user. Figure 11B [33] showcases how different colors and graphical elements can be used to enhance the explanation of the model’s outcomes and ultimately support the user in managing their stress levels effectively [33]. Another notable application of multimodal explainability lies in providing personalized recommendations to end users. Researchers introduced a multimodal explainability approach in a study investigating the relationship between blood pressure and lifestyle factors [38]. This approach generates a report summarizing the relevant blood pressure features and offers tailored lifestyle modifications to improve blood pressure. An example of such a report can be observed in Figure 11C [38], which combines textual and graphical elements to present comprehensive and actionable information to the user. By using user-friendly graphics, visual representations, and multimodal explanations, researchers aim to optimize end-user understanding, engagement, and decision-making processes. These approaches leverage familiar and intuitive formats to convey complex information effectively, empowering users to monitor their health, manage stress, and make informed lifestyle choices.

#### Stage of Explainability

In this systematic review, the application of different explainability methods in health care monitoring varied, with some methods being more extensively explored than others. A total of 88% (22/25) of the reviewed studies used post hoc methods, whereas only 12% (3/25) used ante hoc methods. Model-agnostic methods were used in 60% (15/25) of the reviewed studies, and the rest were distributed between specific-domain (3/25, 12%), attention-based (3/25, 12%), gradient-based (3/25, 12%), and general explainability (1/25, 4%) methods, as seen in Table 5. Among the model-agnostic methods, SHAP was the most widely applied method, appearing in 48% (12/25) of the studies. SHAP aims to provide interpretable explanations for individual predictions made by complex ML models [28]. This also highlights its prevalence as a post hoc method in explainability research. In contrast, only 4% (1/25) of the studies explored an ante hoc explainability approach, specifically the explainable gradient boosting method. This method involves modifying or enhancing the original Extreme Gradient Boosting algorithm to enhance interpretability, indicating a limited use of ante hoc techniques in the reviewed studies.

**Table 5:** Distribution of explainability methods.

	Studies, n (%)
Model-agnostic methods	15 (60)
Specific-domain methods	3 (12)
Attention-based methods	3 (12)
Gradient-based methods	3 (12)
General model explanation methods	1 (4)

^a^Not applicable.

Gradient-based methods garnered attention in 12% (3/25) of the studies, encompassing explainable gradient boosting [43], deep learning important features [58], and layer-wise relevance propagation [45], with each technique being featured once. Explainable gradient boosting represents an advanced iteration of the gradient boosting algorithm, integrating mechanisms to render transparent insights into its decision-making process and feature significance [59]. Deep learning important features, on the other hand, serves as a technique that dissects a neural network’s output prediction for a specific input, unraveling the contributions of all network neurons to each feature of the input [60]. Layer-wise relevance propagation, functioning as a framework, facilitates the deconstruction of deep neural network predictions on a sample into relevance scores [61]. These methods collectively serve the purpose of unveiling the significance of individual features and comprehending the intricate decision-making of complex models. While their numbers are limited, these techniques remain instrumental in providing invaluable insights into model behavior.

Attention-based methods were also studied in 12% (3/25) of the studies, involving interpretable LSTM-attention [46], CAM [47], and interpretable recurrent neural network [40]. In the case of interpretable LSTM-attention, an attention model is used to assign varying weights to input features of financial time series at each time step. This attention feature is then used to effectively select relevant feature sequences for input into the LSTM neural network, aiding prediction in subsequent time frames [62]. Meanwhile, CAM is an explainability technique used to identify significant regions within an input image that contribute to specific class predictions within CNNs [63]. Furthermore, the concept of an interpretable RNN emerges as a variant of the RNN architecture aimed at providing transparent insights into its decision-making process and internal representations, thereby enhancing its interpretability [64]. These methods excel in capturing salient regions and identifying sequential dependencies in health care data. Their use is driven by the need to understand how the model focuses on specific areas or patterns when making predictions.

Specific-domain methods such as GNNExplainer for graph neural networks and temporal summaries for time-series data were investigated in 12% (3/25) of the studies [47,48,56]. These methods cater to the unique characteristics of specific domains, allowing for domain-specific insights. Temporal summaries, in particular, were applied twice [56,57], emphasizing the significance of time-series data in health care monitoring. Finally, general model explanation methods were explored in a single study [65]. This broader category encompasses various techniques, but its limited application in this review suggests that researchers focused more on domain-specific or model-specific approaches in health care monitoring. The variation in the number of studies that applied each method indicate the varying levels of interest and emphasis placed on different explainability techniques. Researchers may choose certain methods based on their effectiveness in providing understandable explanations, compatibility with the data type, or the specific requirements of the health care monitoring domain being studied.

#### Scope of Explainability

This review encompassed studies using 15 local explainability methods and 10 global explainability methods applied in the context of health care monitoring. The inclusion of a larger number of local explainability methods, accounting for 60% (15/25) of the studies, indicates the significance of understanding individual predictions and the specific factors influencing them. These methods provide granular insights into model behavior and help build trust by explaining the rationale behind individual decisions. However, the presence of global explainability methods is also noteworthy as they offer a broader perspective on model behavior, identifying general patterns and highlighting features that consistently contribute to predictions across the entire dataset. The balance between local and global explainability methods in this review demonstrates the importance of both instance-level interpretability and a comprehensive understanding of model behavior in the context of health care monitoring. Researchers recognize the need for a multifaceted approach to ensure transparency, reliability, and generalizability in interpreting the outcomes of health care models.

#### Problem Types Addressed by AI Models

Overall, classification emerged as the most prevalent underlying problem in the reviewed studies, accounting for 52% (13/25), followed by regression at 28% (7/25). In addition, 4% (1/25) of the studies used a rule-based approach [56], whereas another (1/25, 4%) used a model explanation [65] as the underlying problem type. It is noteworthy that the combined total of regression and classification studies exceeded the total number of reviewed papers (N=25). This disparity arises from certain studies using models for both classification and regression tasks (3/25, 12%). The predominance of classification problems (16/25, 64%) in the reviewed studies indicates the significance of accurately categorizing health care data for diagnostic, predictive, or decision-making purposes. Gradient boosting emerged as the most frequently used algorithm for classification (5/25, 20%), highlighting its effectiveness in achieving high predictive performance and capturing complex relationships within health care datasets. Support vector machine (SVM) was also commonly used (3/25, 12%), known for its ability to handle both linear and nonlinear classification problems [66]. SVM generally exhibits improved performance when applied to smaller datasets [67]. In addition, decision trees, Neural Structured Learning, graph neural networks, and CNNs were selected for specific classification tasks (5/25, 20%), reflecting the diversity of approaches used to tackle different health care monitoring challenges.

**Figure 12:**
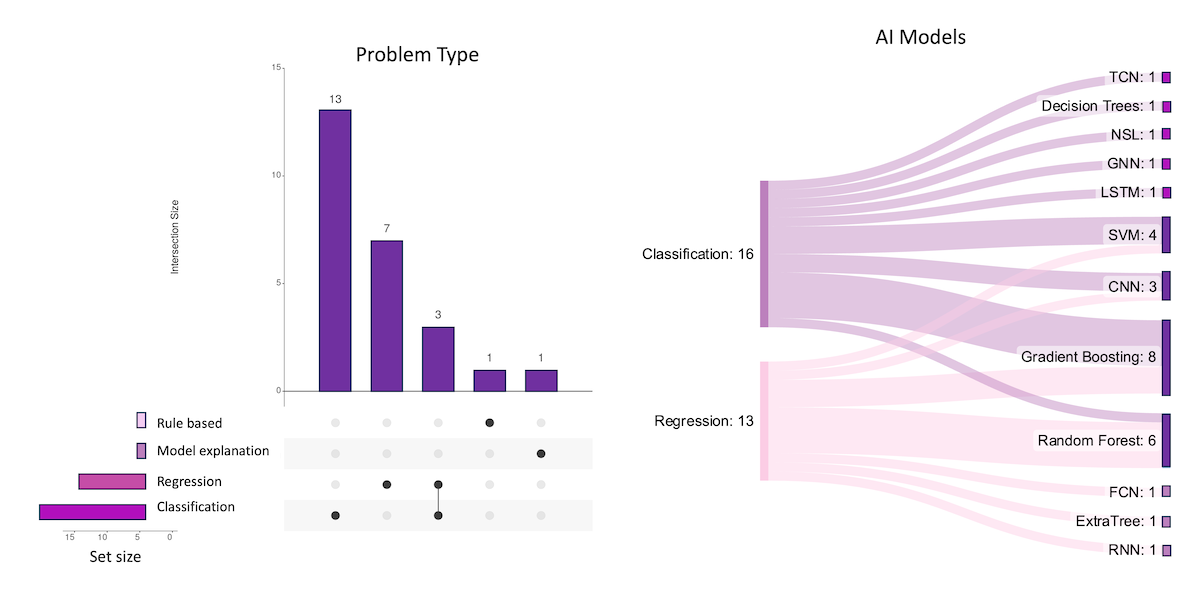
(A) Overview of problem types in the reviewed papers; (B) regression and classification problem types with the corresponding machine learning or deep learning model application. CNN: convolutional neural network; FCN: fully convolutional network; GNN: graph neural network; LSTM: long short-term memory; NSL: neural structured learning; RNN: recurrent neural network; SVM: support vector machine; TCN: temporal convolutional network.

In regression problems (13/25, 52%), the reviewed studies used various algorithms to predict continuous or numerical outcomes relevant to–health care. Gradient boosting, known for its powerful ensemble learning capabilities, was the most prevalent algorithm (3/25, 12%), indicating its effectiveness in capturing nonlinear relationships and providing accurate regression predictions. Random forest (5/25, 20%) and SVM (1/25, 4%) were also used for regression tasks, leveraging their ability to handle complex datasets and capture intricate relationships between input features and target variables. Furthermore, CNNs, fully convolutional networks, ExtraTree, and RNNs were each used in specific regression studies (4/25, 16%), showcasing their suitability for capturing temporal or spatial patterns and making accurate predictions in health care monitoring contexts. It is worth noting that decision trees are generally considered more interpretable or transparent compared to some other ML models. The distribution of problem types observed in this review reflects the complexity and diversity of health care monitoring tasks (Figure 12). By leveraging these algorithms effectively, researchers can develop robust and accurate health care–monitoring models that cater to different types of problems.

### Training Dataset

In the development of AI models, they can be constructed using either user datasets, which are derived from real-world user interactions and activities and capture user behaviors and characteristics, or benchmark datasets, which are widely recognized standards for comparison and evaluation within the field [68]. Irrespective of the dataset used to train the AI model, some explainability models were evaluated with end users, whereas others were not. As the primary goal of explainability is to enhance user interaction with AI models, real-world evaluation involving lay users is essential.

Table 6 provides an overview of the datasets used in training AI models for health care monitoring, where 80% (20/25) of the studies used user datasets in comparison to 20% (5/25) of the studies, which used a benchmark dataset. Researchers adopted various strategies when selecting these datasets. Some chose to create custom datasets gathered by having users wear devices such as the Empatica E4 wristband to capture physiological signals while performing specific tasks. Conversely, others used benchmark datasets, exemplified by the MyFitnessPal food log dataset, comprising 587,187 days of food log data across 9000 users for 180 days [69]. The decision to use benchmark datasets often stems from resource constraints, such as limited time or access to participants. Conversely, the creation of custom datasets was preferred when specific testing conditions such as unique population groups or environments were not covered by existing datasets. Of the 25 studies reviewed, 20 (80%) opted to construct models using a dataset created by the authors, whereas 5 (20%) chose established benchmark datasets, such as the Floodlight Proof-of-Concept dataset used in the study by Creagh et al [45], the MyFitnessPal dataset used in the study by Harris et al [56], the MHEALTH dataset used in the study by Uddin and Soylu [49], the Continuous Glucose Monitoring Intervention in Teens and Young Adults With Type 1 Diabetes study dataset [70], and the Human Activity Recognition database [71].

An analysis of the experimental setups for training datasets revealed distinct approaches, as shown in Table 6. It is noteworthy that the evaluation environment, whether conducted in the real world or in a controlled setting, plays a role in shaping the evaluation process. In this review, data collection occurred in real-world settings in 80% (20/25) of the studies, reflecting the natural environmental conditions in which users typically interact with the technology, such as registering the food they consume in a day [56]. Conversely, 20% (5/25) of the studies opted for a controlled environment for their data collection. This controlled setup involves specific conditions designed to eliminate external influences and noise. For instance, a study collected gait signals using sensors embedded in shoe insoles along a 27-meter straight corridor, ensuring a controlled and consistent testing environment [50]. In addition, physiological signals such as photoplethysmography and EDA were gathered using wearable wristbands such as Empatica E4 or Samsung Gear within soundproof rooms, eliminating auditory and visual distractions [42]. In another instance, during a private study, EDA and HR signals were collected using Empatica E4 devices within the controlled environment of a hospital setting [58]. After training the model, some studies went a step further and tested the explainability model with lay users. Of the 25 studies, only 5 (20%) tested the explainability output with potential end users. The Human-Centered Evaluation section explores the different aspects of the human-centered evaluation methods.

**Table 6:** Experimental setup of the user and benchmark datasets.

Training dataset	Experimental setup, n (%)		Total datasets, n (%)
	In the wild	Controlled	
User dataset	>16 (64)	4 (16)	20 (80)
Benchmark dataset	5 (20)	0 (0)	5 (20)
Total experimental setup	20 (80)	5 (20)	—^a^

^a^Not applicable.

### Human-Centered Evaluation

#### Overview

The following sections focus on the 20% (5/25) of the studies (Table 7) included in this review that evaluated the user perception of explainability. They examined key aspects, such as the studied population, materials and methods, study duration, design, medium of interaction, and type of interaction. Table 7 summarizes the results of the 20% (5/25) of user studies.

**Table 7:** Human-centered evaluation of the explainability model.

Study	Studied population	Materials	Study duration	Study design	Method	Medium of interaction	Type of interaction
Kim et al [33]	Healthy participants	Interviews, questionnaire, and use logs	30-min introductory session, 25-day MindScope use, and 50-min follow-up interview	Pretest-posttest study	Mixed methods	Apps	Interactive
Wozniak et al [55]	Healthy participants	Survey	3 min 35 s to 5 min 43 s	Between subject	Mixed methods	Apps	Passive
Leitner et al [39]	Patients with prehypertension or hypertension	Survey and data logs	6 mo	Between subject	Quantitative	SMS text messages	Passive
Harris et al [56]	Healthy participants	Survey	Not mentioned	Within subject	Quantitative	No instruments	Passive
Chiang et al [38]	Patients with hypertension	Data log	1 mo	Between-subject and pretest-posttest study	Quantitative	Emails	Interactive

#### Studied Population

In all the studies (5/5, 100%), the primary focus was on providing explainability to end users who were not medical experts, researchers, or AI experts. The emphasis was on ensuring that the explainability methods used were simple and easily understandable for the target audience. Of these 5 studies, 3 (60%) specifically targeted healthy individuals [33,55,56]. The focus of these studies was on monitoring and addressing aspects such as stress levels, promoting trust in AI-generated step goals, and providing fitness summaries. The goal was to provide simple and interpretable explanations to help individuals monitor and improve their overall well-being. The remaining studies focused on patients with stage-1 hypertension [38,39], which is when the blood pressure of an individual ranges from 130 to 139 mm Hg systolic or 80 to 89 mm Hg diastolic most of the time. In these studies, the primary objective was to provide explainability for lifestyle recommendations aimed at managing and improving blood pressure. The goal was to empower patients with understandable explanations regarding the recommended lifestyle changes, facilitating their active participation in their health management. While many explainability papers center on creating models for clinicians, such as opioid detection [35] and multiple sclerosis ambulatory characteristics [45], none of the reviewed studies specifically addressed this target audience. This observation highlights a gap in the current research landscape as there is potential for further exploration and development of explainability models tailored to the needs and understanding of clinicians and other health care professionals.

#### Instruments for Delivering Explainability

In the reviewed studies, explainability was presented and communicated to the users through various instruments and platforms. Most of the studies (7/25, 28%) opted for a mobile app to deliver the explainability components to the users [33,39,55]. Only 20% (1/5) of the studies used email as the medium for presenting the explainability [38]. This suggests that alternative communication channels can be used to deliver explainability, such as computers or wearable wristbands, depending on the specific study context and target audience. By using different instruments for delivering explainability, the researchers demonstrated their flexibility in tailoring the presentation of information to suit the needs and preferences of users.

#### Type of Data Collected

In the user studies, various types of data were collected to gather insights and perspectives from the participants. These methods included interviews, questionnaires, and use logs, which provided qualitative and quantitative information about user experiences, preferences, and use patterns [33]. In addition, surveys were used to gather structured feedback and opinions from the participants [55,56]. Examples of surveys include the Perceived Stress Scale [72] used in the study by Kim et al [33], the Goal Commitment Scale [73], and the Trust Scale [74] used in the study by Wozniak et al [55]. In one study, a combination of surveys and data logs was used to capture both subjective responses and objective use data [39]. Certain studies (1/5, 20%) also used data logs to record and analyze user interactions and behavior [38]. It is worth noting that this diverse range of data collection methods reflects the adaptability of researchers in selecting appropriate tools for evaluating explainability models, with no discernible pattern emerging from the reviewed studies in terms of preferred data types.

#### Duration of the Study

The data collection duration in the user studies varied. In one study, the time for data collection per participant ranged from 3 minutes and 35 seconds to 5 minutes and 43 seconds, indicating brief interactions or tasks [55]. Another study involved a 30-minute introductory session followed by a 25-day MindScope use study and concluded with a 40-minute follow-up interview, allowing for a more extensive examination of participant experiences [33]. In addition, data collection occurred over 1 month in a specific study [38], providing a medium-term perspective on user engagement. One study spanned a longer duration of 6 months [39], enabling researchers to comprehensively assess user experiences and outcomes over an extended period. These variations in data collection duration highlight the diverse approaches used in user studies and provide insights into the range of participant engagement and the depth of data collection achieved in each study.

#### Study Design

The user studies incorporated various study designs to evaluate the effectiveness of the explainability models. These designs included pretest-posttest studies where data were collected before and after participant interactions with the system to assess any changes or improvements [33]. Between-subject and between-subject vignette designs involved dividing participants into different groups to compare the impact of different conditions or scenarios [39,55]. Within-subject designs allowed participants to experience multiple conditions or treatments, enabling comparisons within individual contexts [56]. In addition, between-subject designs and pretest-posttest studies were combined in some studies (1/25, 20%) to evaluate the system’s impact across different groups comprehensively [38]. The review should reflect a clear preference or consistent pattern in the selection of a specific study design. However, the choice of study design seems to be driven by the researchers' individual preferences and the specific objectives of their investigations.

#### Challenges Reported

Researchers reported several challenges while conducting user studies. One notable issue pertained to the experimental design, wherein participants had limited interaction with each explanation type, restricted to 5-day intervals [33]. This abbreviated time frame may not have been sufficient to adequately assess the varying impacts and distinctions among different explanation methods. To address this limitation, a longitudinal study could be considered to offer a more comprehensive understanding. Another challenge centered on the need for a control group [33]. The inclusion of a control group would enable a clearer differentiation of the effects of prediction and explanation on stress reduction and management outcomes. Moreover, the task of counterbalancing the diverse explanations also posed a challenge [33]. Ensuring that each participant receives explanations in a balanced manner could enhance the validity of the findings. Furthermore, some studies (2/5, 40%) had a restricted participant demographic, such as solely involving college students [33] or relying on a single platform such as Mechanical Turk [75] for data collection [55]. In addition, some studies (2/5, 40%) had to contend with a limited number of participants [38,39]. These challenges collectively underline the importance of carefully addressing methodological constraints to ensure the robustness and generalizability of the study’s conclusions.

### Technologies for Capturing Quantified Self Data

**Figure 13:**
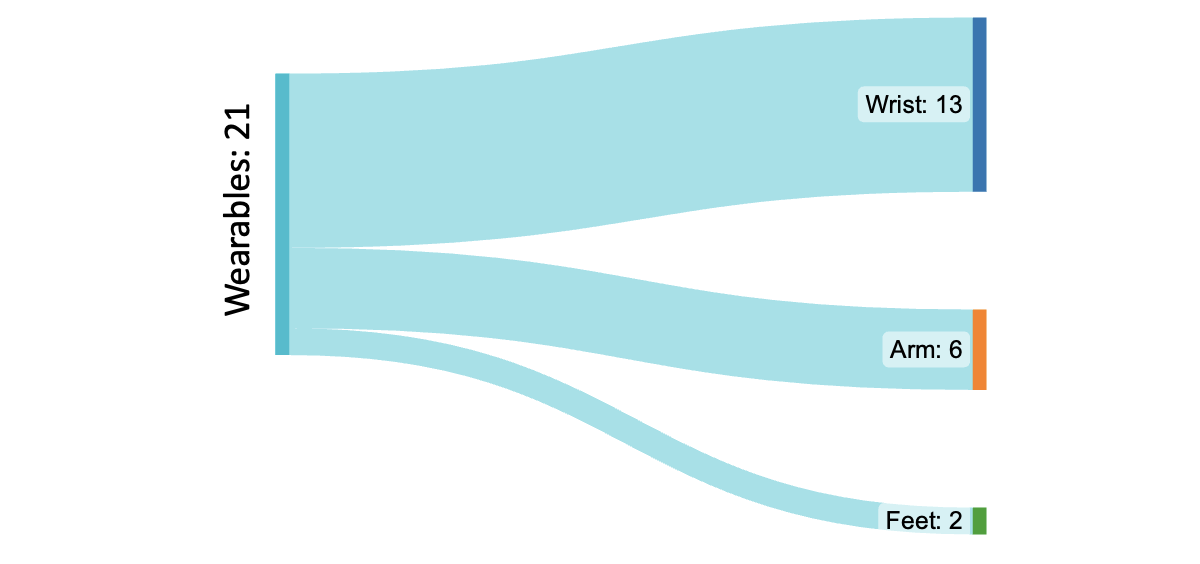
Sources of the data collected.

**Figure 14:**
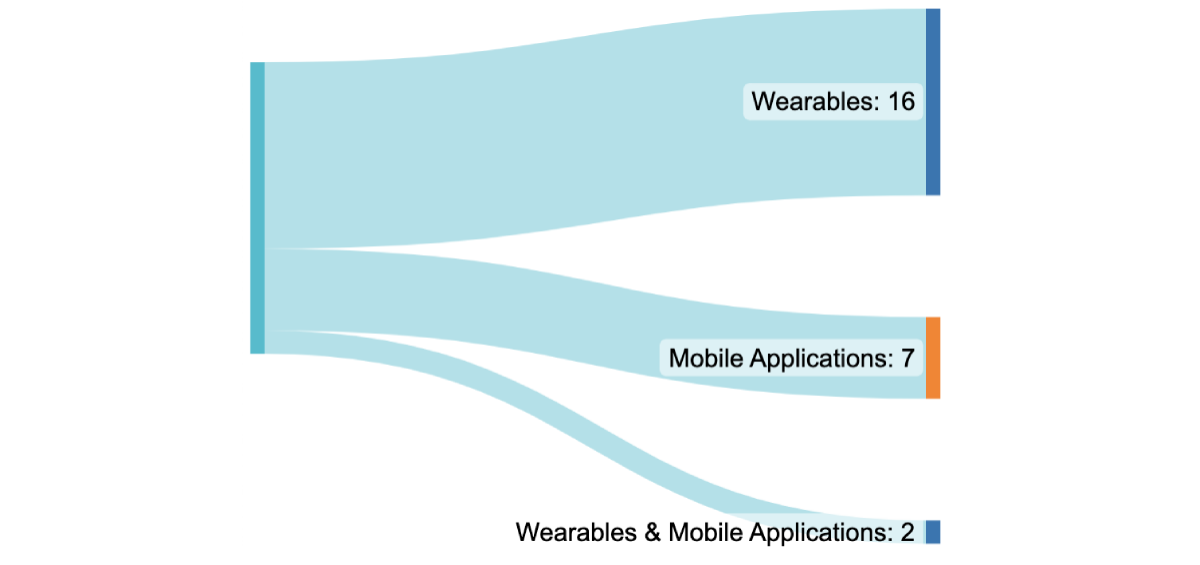
Data collection medium.

Among the wearable devices reviewed, the wrist was the most common placement location (13/25, 52%), as illustrated in Figure 13. Some studies (4/25, 16%) used commercially available devices such as Fitbit and Galaxy Watch [43,46,48,55], which gather data such as step count, distance traveled, calories burned, weight, HR, sleep stages, active minutes, and even location. On the other hand, research-based wristbands such as the Empatica E4 focus on capturing data such as EDA, blood volume pulse, HR, and interbeat interval [35,37,58]. In addition, wearables were predominantly utilized in the studies (16/25, 64%), while mobile applications were used in a smaller proportion (7/25, 28%), as shown in Figure 14.

In addition, other wearables were identified, specifically arm-based devices (6/25, 24%) such as glucose monitoring devices (eg, Freestyle Libre and BioStampRC) [2,41]. These devices serve the purpose of monitoring glucose levels. Furthermore, arm-based devices were also found to collect blood pressure data, with the Omron EVOLV wireless blood pressure monitor being one notable example [39]. In a couple of studies (2/25, 8%), the researchers explored the use of sensors embedded within the shoe insole to capture quantified self data [50,51]. This approach allowed for the collection of data related to foot movement and pressure distribution. In several other studies (7/25, 28%), the focus shifted toward using mobile apps for gathering quantified self data [40,47,52,56,57]. These apps often leveraged GPS data and logs to obtain information about user activities and movement patterns. Overall, this systematic review revealed a wide range of technologies used to capture quantified self data, with wristbands, arm-based devices, shoe insole sensors, and mobile apps playing prominent roles in the collection process.

## Discussion

### Principal Findings

#### Overview

The emergence of wearable technologies has revolutionized the field of health care, enabling the collection of vast amounts of quantified self data and opening new possibilities for AI models. However, the inherent “black box” nature of AI models has hindered their seamless integration into medical practices and public acceptance. This emphasizes the critical role of explainability in bridging the gap between health care professionals and AI experts. In this review, we delved into the adoption of explainable models in health care, with a specific focus on wearable technologies and quantified self data. The following sections provide a comprehensive exploration of the 3 main themes: wearables, explainability, and human-centered evaluation.

#### Wearables and Data

To address RQs 1 and 2, the widespread application of wearables in the health care field has been extensively explored in various reviews [20,76]. These wearable applications encompass a diverse range, from assessing wearables’ usability [77] to using wearable cameras for self-management [78], revealing their limitless potential. However, despite their prevalence and versatility, the aspect of explainability in wearable technologies has received less attention. Previous reviews on wearables in health care have attracted the interest of researchers, covering a wide range of research papers. Reviews focusing on wearables have yielded many papers, such as 82 [20] or 73 [79] reviewed papers, whereas reviews on explainability have produced even more extensive results, such as 91 [13], 93 [80], or 196 [81] reviewed papers. In contrast, this review specifically delved deeper into the investigation of explainability in wearables, and thus, the number of relevant results decreased significantly, with only 25 papers. This discrepancy highlights the relatively limited focus on explainability within the realm of wearable technologies despite their vast potential and relevance in the health care domain. Notably, explainability is particularly vital for the lay public and health care professionals, who form the primary target audience for wearable devices.

Despite the limited focus on explainability in wearables, this review revealed that some of the findings align with general trends observed about wearables. Notably, wrist-worn wearables have emerged as the most commonly used technology, as seen in the Technologies for Capturing Quantified Self Data section, which is in line with previous research [82,83]. Specifically, Fitbit and Empatica E4 wristbands were identified as the most used wearables, with Fitbit being more dominant than Empatica E4 in the literature [82,83]. This can be due to the high cost of Empatica E4 in comparison to Fitbit devices [84] as it costs an average of US $1700, whereas the Fitbit Sense costs US $170, which is 10 times cheaper. However, the reason for choosing a specific wearable depends on the use case and aim of the study as some signals are not captured by Fitbit, such as those from temperature sensors and accelerometers. For example, the Empatica E4 wristband stands out for its promising potential in the unobtrusive measurement of HR variability [85]. The data collected from these wearables predominantly consisted of physiological signals, making them the most used input data for developing AI models. User-generated datasets significantly outnumbered benchmark datasets. When examining the experimental setups, it became evident that most studies (20/25, 80%) operated within uncontrolled real-world settings. A recent review underscored the constraints associated with imbalanced datasets primarily stemming from publicly available sources rather than real-world data [13], which is contrary to the findings of this review.

While wearables in health care have shown immense promise, there are critical considerations and recommendations to ensure their effective use while aligning with the goals of explainability. First, there is an urgent need to prioritize the development of XAI models and user interfaces for wearables. This is essential to bridge the gap in understanding between users and the insights derived from wearable data, thereby enhancing trust, reliability, and usability—key components of explainability. Second, when choosing wearables for health care applications, researchers and practitioners must carefully assess their specific data requirements against factors such as cost, comfort, and data capture capabilities. This strategic selection of wearables can significantly impact the quality and relevance of collected data, contributing to transparency and fairness in data collection. Moreover, it is crucial to continue emphasizing the use of real-world data in wearable studies. Such data better reflect the complexity of health care scenarios and enhance the practicality of research findings, ensuring that causal relationships and privacy concerns are appropriately addressed. By adhering to these recommendations and aligning them with the goals of explainability, we can ensure that wearable technology in health care not only realizes its potential but also serves users effectively, ethically, and transparently.

#### Explainability for Wearable Data

To address RQs 3 and 4, various explainability methods were explored, and a noticeable trend emerged toward using model-agnostic approaches (15/25, 60%) such as SHAP, as seen in the Stage of Explainability section. Model-agnostic methods have gained popularity due to their ability to be applied to a wide range of ML models regardless of complexity or type, unlike other methods such as gradient-based or attention-based methods. These methods provide explanations independently of the model’s internal workings [86]. Interpretable models face a trade-off between accuracy and interpretability [87]. Model-agnostic interpretability treats the model as a black box, creating a separation between interpretability and the model. This approach allows the model to be flexible and versatile, accommodating diverse ML methods, including complex ones such as deep neural networks [86]. Decoupling interpretability from the model enables a balance to be achieved between accuracy and interpretability, providing a valuable tool for understanding and explaining the model’s behavior [86]. Furthermore, this approach empowers medical professionals to gain valuable insights into the decision-making process and understand the impact of different features on predictions for conditions or diseases. By understanding how the AI models arrive at their conclusions, medical professionals can build trust and confidence in using AI technology [88].

SHAP stands out as a widely used model-agnostic method (12/25, 48%), offering feature importance values based on the Shapley value from the cooperative game theory [28]. This framework helps interpret the impact of individual features on model predictions [28]. The trends in explainability identified in this review align with previous findings in the literature. A previous review categorized explainability into different types, such as feature, example-based, and textual explanations [89]. Feature explainability involves techniques such as SHAP, LIME, and CAM. In this review, a similar pattern emerged, with feature explainability being more prevalent. However, the choice of explainability method depends on the specific use case, the model in question, and desired interpretability levels. While model-agnostic methods such as SHAP have gained popularity, other techniques such as rule-based models, decision trees, and feature importance analysis may be more suitable for certain scenarios.

In this review, it was observed that post hoc explainability methods were more commonly used (22/25, 88%) than ante hoc explainability methods (3/25, 12%) in the field of wearables, as seen in the Stage of Explainability section. This trend is attributed to the fact that post hoc methods can be applied to any existing model regardless of its initial explainability capabilities. On the other hand, ante hoc methods require models to be designed and trained with explicit explainability mechanisms, making these methods less prevalent. These findings align with those of previous studies, which have reported a higher prevalence of post hoc methods [81,89]. However, compared to reviews generally focusing on explainability, ante hoc methods are used more frequently than post hoc methods [26,90]. This discrepancy may be attributed to different research contexts and objectives.

Furthermore, it was observed that most of the explainability methods reviewed in this study had a local scope (15/25, 60%) rather than a global one (10/25, 40%), as seen in the Scope of Explainability section. Local explainability methods focus on providing explanations for individual predictions or instances, allowing for a detailed understanding of how the model arrived at a specific decision. On the other hand, global explainability methods aim to provide insights into the model’s overall behavior and decision-making process. The prevalence of local explainability methods in this review suggests a more targeted approach to understanding specific model predictions, which can be valuable in practical applications in which users may be more interested in individual predictions rather than a holistic view of the entire model. When discussing local and global explainability, it is valuable to consider and present both types together rather than focusing on only one [91].

The explainability of models was often conveyed through visual formats (17/25, 68%) using different types of graphs. Visual explainability has demonstrated greater prominence when compared to other outputs such as numerical, rule-based, and textual explanations [26,81,92]. This preference for visual representations highlights the effectiveness of using visualizations to enhance the interpretability and understanding of complex AI models. The level of explainability output is linked to the end-user audience. For instance, when the end user comprises the public, the output is tailored to be visually engaging, interactive, and user-friendly, as seen in the Multimodal Explanations section. In contrast, when the intended audience consists of researchers or medical professionals, the explanation may be presented through more technical means, such as data-rich visualizations (eg, heat maps), as seen in the Visual Explanations section.

Model explainability is paramount in the evolving landscape of AI research. The decisions in this realm are critical. Model-agnostic methods, notably SHAP, have garnered attention for their adaptability. A significant proportion of studies (24/25, 96%) adopted post hoc explainability as it offers the convenience of integration after model development. While there is a discernible tilt toward elucidating local predictions, a holistic approach encompassing both local and global perspectives is essential. Visual presentations stand out as the preferred mode to enhance the comprehension of AI models. From the findings, given that the datasets used in building the AI models were mostly from users (20/25, 80%) rather than benchmark datasets (5/25, 20%) and the emphasis on using local (15/25, 60%) rather than global (10/25, 40%) explainability, this showed a distinct move toward personalization. This shift is anticipated given the growing momentum of precision medicine on the global stage whether in research or practice and the emphasis on the importance of patient-centered health care.

#### Human-Centered XAI Needs More User-Based Evaluation

To address RQ 5, the primary objective of explainability is to create models that are transparent and understandable for end users, making it crucial to involve them in the validation process. However, in this review, of the 25 papers analyzed, only 5 (20%) reported user studies, indicating a limited focus on user evaluations. This trend aligns with findings in the broader literature, where only 1 in 5 papers included user evaluations [26]. Furthermore, the user evaluations conducted in the reviewed studies primarily targeted healthy individuals and patients with hypertension, limiting the representation of other populations. Despite the diversity of domains in the studies, the scope of user evaluation still needs to be narrower. Expanding the range of user evaluations across various populations is essential to enhance the real-world applicability and acceptance of AI models in the health care domain.

Assessing the explainability of wearables is crucial. As wearables rapidly evolve into essential tools for persistent health monitoring, they produce massive amounts of data daily. These complex data necessitate clear interpretation to make well-informed decisions [93]. As these devices become more woven into a person’s daily life, the decisions based on their data significantly affect health and well-being. Such decisions are deeply personal. Hence, it is imperative not just to have accurate data interpretation but also for users to easily understand, use, and trust the results. If users, including medical professionals, cannot comprehend or have confidence in these insights, they might hesitate to act on them or could make incorrect choices.

Considering the personal and intimate nature of data from wearables, user studies are essential. Furthermore, gauging XAI’s effectiveness with users is paramount to foster trust, customize explanations to the users’ needs, understand the users’ context, and ensure overall usability [93]. This assessment not only supports and evaluates the alignment with AI’s ethical and regulatory standards but also enhances its overall acceptability. However, in-the-wild user studies remain a notable gap in this area [94]. The design, development, and evaluation of AI and ML systems must transition to real-world applications. This shift calls for interdisciplinary collaboration and multiple design and evaluation iterations [95]. While real-world evidence is beneficial for patients, caregivers, medical professionals, and society at large, adopting a human-centric approach can be challenging due to the sociotechnical aspects involved [53].

### Limitations

While our literature search was comprehensive, the application of strict inclusion and exclusion criteria resulted in a relatively low number of studies being included in the review. We also stopped the collection and started the analysis and reporting work on December 31, 2022. This selection process, while ensuring the quality of the included studies, may limit the generalizability of our findings to a broader population of studies. In addition, the limited number of studies involving end users in our review underscores the need for caution when concluding this subset of the data. To address these limitations and enhance the robustness of the findings, further research with more extensive and diverse samples is recommended.

### Implications for Practice and Future Development

The findings of this review have several implications for both current practice and future development in the field of XAI in wearable technologies for health care. For practice, this review highlights the importance of incorporating explainability into the design and implementation of wearable technologies used in health care. As these technologies become more prevalent in medical settings, medical professionals and end users must understand how AI models arrive at their decisions. By providing interpretable and transparent explanations, medical professionals can gain insights into the decision-making process of complex models and build trust and confidence in using AI technology. This, in turn, can lead to more effective and informed decision-making in patient care and treatment.

Moreover, this review emphasizes the need for more user evaluation and involvement in the development of XAI models. Understanding the perspectives and needs of end users is essential to ensure that the explanations provided by the models are meaningful, useful, and user-friendly. Future development should focus on integrating user feedback into the design process, enabling personalized explanations. In addition, this review highlights the significance of visual outputs for presenting explainability. While the current research landscape in XAI primarily relies on visualization and text-based explanations, there needs to be more exploration into alternative modalities for XAI, such as audio or gamification. To advance future development in this field, it is imperative to shift our focus from existing trends toward addressing these gaps. Instead of solely concentrating on established approaches, we should actively investigate novel methods that may offer more effective means of conveying complex model behavior to diverse audiences, including medical professionals and patients. By diversifying our approach to XAI and considering alternatives such as audio or gamification, we can strive to create simpler and more user-friendly explanations that not only foster greater understanding but also enhance the acceptance and adoption of AI technologies in health care.

### Conclusions

This review highlights the nascent and growing significance of XAI in wearable technologies for health care, with 25 research papers included over the last 5 years. The results highlighted that, while wrist-worn wearables such as Fitbit and Empatica E4 are commonly used, the focus on explainability is relatively limited. Post hoc methods such as SHAP emerged as popular choices due to their versatility. Visual outputs are commonly used for user-friendly representations. However, this review highlighted the limitations in user evaluation and the importance of involving users in the process. The small number of human-centered evaluation studies limits the generalizability of the results. Overall, further research in the area of XAI and wearables can pave the way for more transparent and reliable AI models in health care applications.

## Appendix

Multimedia Appendix 1 Search strategy used for the databases.

Multimedia Appendix 2 PRISMA (Preferred Reporting Items for Systematic Reviews and Meta-Analyses) checklist.

Multimedia Appendix 3 Overview of explainability features per reviewed paper.

## Abbreviations

AI: artificial intelligence
CNN: convolutional neural networkDL: deep learning
ECG: electrocardiographyEDA: electrodermal activityHR: heart rate
LIME: local interpretable model-agnostic explanations
LSTM: long short-term memory
MeSH: Medical Subject Heading
ML: machine learning
PRISMA: Preferred Reporting Items for Systematic Reviews and Meta-Analyses
RQ: research question
SHAP: Shapley Additive Explanations
SVM: support vector machine
XAI: explainable artificial intelligence

## Data Availability

All data generated or analyzed during this study are included in this published article (and its supplementary information files).
