# Supplementary material for: Exploring the Applications of Explainability in Wearable Data Analytics: Systematic Literature Review
Source: J Med Internet Res. 2024 Dec 24;26:e53863. doi: 10.2196/53863 (PMC11707450; doi:10.2196/53863)
Supplement: Multimedia Appendix 1 [file jmir_v26i1e53863_app1.docx]

**Appendix A:** Search strategy used for the Databases

| Database | Keywords and search rules |
| --- | --- |
| IEEE | (XAI OR “explainable AI” OR explainab* OR “explainable artificial intelligence”) AND (“machine learning” OR “deep learning” OR interpretab* OR “decision support system” OR recommend* OR “recommender system” OR cdss OR “diagnostic decision support system” OR ddss OR “personal health record” OR “PHR decision support” OR “EHR decision support”) AND (physician* OR clinic* OR patient*) AND (sleep OR “physical activit*”  OR “activity” OR  “exercise” OR “physical exercise”) AND (wearable* OR self-track OR “self track” OR sensor OR “quantified self” OR “self quantified” OR ”quantified-self”) |
| ACM, PubMed, SpringerLink, JMIR,  Nature,  Scopus. | (XAI OR "explainable AI" OR explainab* OR "explainable artificial intelligence" ) AND ( "machine learning" OR "deep learning" OR interpretab* OR "decision support system" OR recommend* OR "recommender system" OR cdss OR "diagnostic decision support system" OR ddss OR "personal health record" OR "PHR decision support" OR "EHR decision support" ) AND ( physician* OR clinic* OR patient*) AND ( sleep OR "physical activit*" OR "physical exercise*" ) AND ( wearable* OR "self-track*" OR "self track*" OR "quantified self" OR "quantified-self" OR "self quantif*" OR "self-quantif*" OR sensor ) |
