# Supplementary material for: Exploring the Applications of Explainability in Wearable Data Analytics: Systematic Literature Review
Source: J Med Internet Res. 2024 Dec 24;26:e53863. doi: 10.2196/53863 (PMC11707450; doi:10.2196/53863)
Supplement: Multimedia Appendix 3 [file jmir_v26i1e53863_app3.docx]

Appendix B: Overview of Explainability Features per reviewed paper (PH: physiological status, A: Activity, S: Sleep, M: Mood, N: Nutrition, O: Other, V: Visual, T: Textual, P: Post-hoc, AH: Ante-hoc, G: Global, L: Local, R: Regression, C: Classification, M: Model explanation, RL: Rule-based)

| Source | Aim | Technology | Model | Input Data | | | | | | Output Format | | Stage | | Scope | | Problem Type | | | |
| --- | --- | --- | --- | --- | --- | --- | --- | --- | --- | --- | --- | --- | --- | --- | --- | --- | --- | --- | --- |
|  |  |  |  | PH | A | S | M | N | O | V | T | P | AH | G | L | R | C | M | RL |
| [3] | To support trainers and coaches in monitoring and managing athletes' fitness activity and results. | FitBit | Counterfactual Explanations |  | A |  | M | N |  |  | T | P |  |  | L |  | C |  |  |
| [44] | To compare between seven wearables for stress detection. | Empatica e4, Samsung Gear, etc. | SHAP | PH (PPG, EDA, ACC, etc.) |  |  |  |  |  | V |  | P |  | G |  |  | C |  |  |
| [40] | To investigate BP-lifestyle relationships and provide personalized recommendations. | Samsung Galaxy Watch, Wireless BP monitor | SHAP | PH  (BP, HR) | A | S |  |  |  | V | T | P |  | G |  | R |  |  |  |
| [51] | To differentiate between healthy and people with multiple sclerosis. | Mobile phone and Smartwatches | LRP | PH (ACC) |  |  |  |  |  | V |  | P |  |  | L |  | C |  |  |
| [56] | To recognize influenza-like symptoms. | Mobile Application | GNN interpretability + CAM |  | A (Mob) |  |  |  | Social Interactions | V |  | P |  |  | L |  | C |  |  |
| [2] | To predict hypoglycemia and hyperglycemia with a lead time of up to 60 minutes. | CGM | SHAP | PH (BG) |  |  |  |  |  | V |  | P |  | G |  |  | C |  |  |
| [45] | To detect COVID-19 infection and explain feature importance and post-test behavior. | FitBit | Explainable Gradient Boosting | PH (HR) | A | S |  |  |  | V |  |  | AH | G |  |  | C |  |  |
| [38] | To detect opioid use utilizing physiological signals from a wrist-mounted sensor. | Empatica E4 | Attribution-based explainable AI | PH (HR, TEMP, IBI, EDA, ACC) |  |  |  |  |  | V |  | P |  |  | L | R | C |  |  |
| [75] | To examine the effect of a new therapeutic on BP | Mobile phone | SHAP | PH (BP + weight) |  |  |  |  | Demographics | V |  | P |  |  | L |  | C |  |  |
| [42] | To predict weight change. | Mobile Application | Interpretable RNN | PH (weight) |  |  |  | N | Exercise log | V |  | P |  |  | L | R |  |  |  |
| [50] | To explore the link between peripheral and central autonomic bio signals using SEEG. | Empatica E4 and SEEC electrodes | DeepLIFT | PH  (SEEG, EDA, HR, PPG) |  |  |  |  |  | V |  | P |  |  | L | R |  |  |  |
| [49] | To produce numeric-to-text summaries. | Mobile phone | Temporal Summaries |  |  |  |  | N |  |  | T | P |  |  | L |  | C |  |  |
| [48] | To generate summaries for users to evaluate health data and compare against general health guidelines. | Mobile Phone | Temporal Summaries |  |  |  |  | N |  |  | T | P |  |  | L |  |  |  | RL |
| [69] | To screen for sarcopenia using gait signal parameters. | Embedded sensor in show insole | SHAP | PH (gait) |  |  |  |  |  | V |  | P |  |  | L |  | C |  |  |
| [74] | To analyze the inertial-sensor-based gait. | Embedded sensor in show insole | SHAP, LRP | PH (gait) |  |  |  |  |  | V |  | P |  |  | L |  | C |  |  |
| [41] | To generate personalized lifestyle recommendations for each patient’s blood pressure (BP). | Mobile Application, Samsung Galaxy Watch, Wireless BP monitor | SHAP | PH (HR) | A | S | M | N |  |  | T | P |  | G |  | R |  |  |  |
| [96] | To predict user-specific health risks using mobility metrics. | Mobile Phone | SHAP |  | A (Mob) |  |  |  |  | V |  | P |  |  | L | R |  |  |  |
| [39] | To detect hypoglycaemia using data collectedhy from smartwatch sensors. | Empatica E4 and Freestyle Libre | SHAP | PH (HR, HRV, BG) |  |  |  |  |  | V | T | P |  |  | L |  | C |  |  |
| [43] | To predict next-day physiological and perceived stress. | ECG sensor and BioStampRC | SHAP | PH (ECG, HR) |  |  |  |  |  | V |  | P |  | G |  | R | C |  |  |
| [55] | To predict sleep efficiency of Insomnia suffers | Fitbit Charge 2 | Interpretable LSTM-Attention |  | A | S |  |  | Demographics | V |  | P |  |  | L | R |  |  |  |
| [46] | To predict user stress levels based on smartphone data. | Mobile Phone (MindScope Application) |  | PH (Acc) |  |  | M |  | GPS data, app usage, environment | V | T | P |  |  | L |  | C |  |  |
| [60] | To predict fluid intake. | Android Smartwatch | GNNExplainer | PH (PPG, ACC, HR, weight) | A |  |  | N | Environment | V |  | P |  |  | L | R |  |  |  |
| [66] | To propose a body sensor-based activity odelling and recognition system | Body Wearable Sensors | LIME | PH (ECG, ACC) |  |  |  |  |  | V |  | P |  |  | L |  | C |  |  |
| [33] | To detect eating moments and to predict and explain glucose levels. | Mobile Application, Abbott FreeStyle, Philips Elan Wristband | SHAP | PH (BG) | A | S |  | N |  | V |  | P |  | G |  | R | C |  |  |
| [47] | To explore effective representations of fitness goals to enhance transparency and build user trust. | Fitbit | Number vs Transparent |  | A |  |  |  |  |  | T | P |  |  | L |  |  | M |  |
